# Skeletal muscle provides the immunological micro-milieu for specific plasma cells in anti-synthetase syndrome-associated myositis

**DOI:** 10.1007/s00401-022-02438-z

**Published:** 2022-05-25

**Authors:** Corinna Preuße, Barbara Paesler, Christopher Nelke, Derya Cengiz, Thomas Müntefering, Andreas Roos, Damien Amelin, Yves Allenbach, Akinori Uruha, Carsten Dittmayer, Andreas Hentschel, Marc Pawlitzki, Sarah Hoffmann, Sara Timm, Sarah Leonard Louis, Nora F. Dengler, Heinz Wiendl, Jan D. Lünemann, Albert Sickmann, Baptiste Hervier, Sven G. Meuth, Udo Schneider, Anne Schänzer, Sabine Krause, Stylianos Tomaras, Eugen Feist, Rebecca Hasseli, Hans-Hilmar Goebel, Laure Gallay, Nathalie Streichenberger, Olivier Benveniste, Werner Stenzel, Tobias Ruck

**Affiliations:** 1 Department of Neuropathology, Institut Für Neuropathologie, Charité – Universitätsmedizin Berlin, Corporate Member of Freie Universität Berlin and Humboldt-Universität Zu Berlin, Virchowweg 15 /Charitéplatz 1, 10117 Berlin, Germany; 2 Department of Neurology with Institute for Translational Neurology, University Hospital Münster, 48149 Münster, Germany; 3 Department of Neurology, Medical Faculty, Heinrich Heine University Hospital Düsseldorf, Moorenstraße 5, 40225 Düsseldorf, Germany; 4 Pediatric Neurology, Faculty of Medicine, University Children’s Hospital, University of Duisburg-Essen, 45147 Essen, Germany; 5 Leibniz-Institut Für Analytische Wissenschaften – ISAS - E.V., 44139 Dortmund, Germany; 6 Centre of Research in Myology, Sorbonne Université, INSERM, Association Institut de Myologie, UMRS 974, 75013 Paris, France; 7 Department of Internal Medicine and Clinical Immunology, Pitié-Salpêtrière University Hospital, 75013 Paris, France; 8 Department of Neurology, Charité – Universitätsmedizin Berlin, Corporate Member of Freie Universität Berlin and Humboldt-Universität Zu Berlin, 10117 Berlin, Germany; 9 Charité – Universitätsmedizin Berlin, Corporate member of Freie Universität Berlin and Humboldt-Universität Zu Berlin, Core Facility Electron Microscopy, 10117 Berlin, Germany; 10 Service de Neuromyologie, GH Pitié-Salpêtrière, University Hospital, 75013 Paris, France; 11 Department of Neurosurgery, Charité – Universitätsmedizin Berlin, Corporate Member of Freie Universität Berlin and Humboldt-Universität Zu Berlin, 10117 Berlin, Germany; 12 Department of Rheumatology, Charité – Universitätsmedizin Berlin, Corporate Member of Freie Universität Berlin and Humboldt-Universität Zu Berlin, 10117 Berlin, Germany; 13 Institute of Neuropathology, Justus Liebig University Giessen, 35390 Giessen, Germany; 14 Friedrich-Baur-Institut, Labor Für Molekulare Myologie, Universität München, 81377 Munich, Germany; 15 Department of Rheumatology, Helios Clinic Vogelsang, Gommern, Cooperation Partner of the Otto-Von-Guericke-University, 39245 Magdeburg, Germany; 16 Department of Rheumatology and Clinical Immunology, Justus-Liebig-University Giessen, Campus Kerckhoff, 61231 Bad Nauheim, Germany; 17 Department of Neuropathology, University Medical Center, 55131 Mainz, Germany; 18 Department of Clinical Immunology, Edouard Herriot University Hospital, 69003 Lyon, France

**Keywords:** Anti-synthetase syndrome, PL-7, PL-12, Jo-1, Plasma cells, Pathophysiology

## Abstract

Anti-synthetase syndrome (ASyS)-associated myositis is a major subgroup of the idiopathic inflammatory myopathies (IIM) and is characterized by disease chronicity with musculoskeletal, dermatological and pulmonary manifestations. One of eight autoantibodies against the aminoacyl-transferase RNA synthetases (ARS) is detectable in the serum of affected patients. However, disease-specific therapeutic approaches have not yet been established.

To obtain a deeper understanding of the underlying pathogenesis and to identify putative therapeutic targets, we comparatively investigated the most common forms of ASyS associated with anti-PL-7, anti-PL-12 and anti-Jo-1. Our cohort consisted of 80 ASyS patients as well as healthy controls (*n* = 40), diseased controls (*n* = 40) and non-diseased controls (*n* = 20). We detected a reduced extent of necrosis and regeneration in muscle biopsies from PL-12^+^ patients compared to Jo-1^+^ patients, while PL-7^+^ patients had higher capillary dropout in biopsies of skeletal muscle. Aside from these subtle alterations, no significant differences between ASyS subgroups were observed. Interestingly, a tissue-specific subpopulation of CD138^+^ plasma cells and CXCL12^+^/CXCL13^+^CD20^+^ B cells common to ASyS myositis were identified. These cells were localized in the endomysium associated with alkaline phosphatase^+^ activated mesenchymal fibroblasts and CD68^+^MHC-II^+^CD169^+^ macrophages. An MHC-I^+^ and MHC-II^+^ MxA negative type II interferon-driven milieu of myofiber activation, topographically restricted to the perifascicular area and the adjacent perimysium, as well as perimysial clusters of T follicular helper cells defined an extra-medullary immunological niche for plasma cells and activated B cells. Consistent with this, proteomic analyses of muscle tissues from ASyS patients demonstrated alterations in antigen processing and presentation. In-depth immunological analyses of peripheral blood supported a B-cell/plasma-cell-driven pathology with a shift towards immature B cells, an increase of B-cell-related cytokines and chemokines, and activation of the complement system. We hypothesize that a B-cell-driven pathology with the presence and persistence of a specific subtype of plasma cells in the skeletal muscle is crucially involved in the self-perpetuating chronicity of ASyS myositis. This work provides the conceptual framework for the application of plasma-cell-targeting therapies in ASyS myositis.

**Supplementary Information:**

The online version contains supplementary material available at 10.1007/s00401-022-02438-z.

## Introduction

The anti-synthetase syndrome (ASyS) is defined as a multiorgan disease affecting mainly skeletal muscle and lung parenchyma (interstitial lung disease), with a characteristic syndromic involvement of other internal organs (e.g., heart), skin (e.g., Raynaud’s phenomenon, mechanic’s hands), and joints [28]. Of note, patients with ASyS harbor one of eight myositis-specific serum autoantibodies directed against the different aminoacyl-transfer RNA synthetases (ARS) that have so far been identified. The most frequent autoantibodies are directed against histidyl-, threonyl- and alanyl-transfer RNA synthetases, called anti-PL-1 (better known as Jo-1), anti-PL-7 and anti-PL-12. Particularly, lung involvement and the specific type of myositis have a considerable impact on quality of life and prognosis in affected patients. Due to limited data from randomized controlled trials, therapy is currently based on empirical usage of nonspecific immunosuppressive drugs [52].

Myositis-specific morphological features of the skeletal muscle affected in anti-Jo-1 associated ASyS were previously identified and characterized, thereby defining a distinct subtype of myositis [4, 47, 49, 68, 73]. These unique features comprise perifascicular necrosis, inflammation with major histocompatibility complex (MHC) expression and myonuclear actin inclusions. Despite progress in our understanding of the morphology and ultrastructural features of ASyS, there remains a significant gap with respect to therapeutic targets and specific therapies that address the underlying disease pathophysiology [5, 68, 73].

The precise role in pathogenicity of ARS autoantibodies in ASyS-associated myositis has not yet been fully elucidated, but there is a clear association of certain disease manifestations including patterns of organ involvement with certain autoantibodies [30, 31, 67]. Anti-Jo-1 is the most common ARS with frequent muscle and joint involvement, whereas anti-PL-7- and anti-PL-12-mediated pathology predominantly affects the lung with a poorer prognosis [46]. Whether the distinct clinical phenotypes are related to distinct pathophysiological features remains largely unknown. Previous pathophysiological studies mainly focused on anti-Jo-1-mediated ASyS. In accordance with the pathogenic role of anti-Jo-1, those studies have shown a correlation between antibody titer and disease activity and no coexistence with other myositis-specific antibodies [29, 48, 69]. Furthermore, Jo-1-induced animal models corroborated certain aspects of the human disease [9, 66]. Interestingly, fragments of amino-acyl tRNA-synthetases also serve as chemokines attracting immune cells to sites of inflammation, further emphasizing their pathogenic role in IIMs. Immunogenicity could potentially originate in the lung where the autoantibodies are expressed in a defined structural conformation then are proteolytically cleaved and become immunogenic [34, 39]. Whether comparable pathways are operative in skeletal muscle has not yet been studied. Autoantibodies are produced by activated plasma cells. In contrast to T cells in the peripheral circulation, plasma cells reside in the bone marrow, and different types of cell and activation states have been linked to autoimmunity [16, 23, 33]. Plasma cells have been detected in tissues with concurrent signs of inflammation in autoimmune diseases such as in salivary glands in Sjögren syndrome [64], synovial fluid in rheumatoid arthritis [53] and in the kidneys of lupus erythematosus patients [43]. More specifically, the plasma cell niche in the bone marrow and a ‘niche’-like home for long-term persistence in other organs has intensely been studied and discussed as the basis of several autoimmune responses in mice and humans [16, 32, 56, 74]. Whether similar pathogenic features can be found in ASyS is unclear.

Here, we study the presence of B cells and plasma cells in the skeletal muscle and in the blood of patients with ASyS. We characterize their activation status and provide a comprehensive description of the immunological milieu that constitutes defining the features of myofibers, fibroblasts and vascular endothelial cells in concert with the analysis of other immune cells. We also investigate potential differences in the immunopathogenic traits of several ASyS subtypes (anti-Jo1, anti-PL7, and anti-PL12). Our findings provide the basis and strongly argue for plasma cell-targeting therapies for this form of myositis.

## Material and methods

### Patients

Clinical data of all patients enrolled in this study are listed in Table 1. Patients were screened in participating centres. Patients were referred to our centres after consultation in primary or secondary care facilities. ASyS patients in this study were required to fulfil current ACR/EULAR criteria for IIM [3]. Further, diagnosis was established by clinical and serological data as well as results from muscle biopsies as recommended in 224^th^ [5] and 239^th^ [45] European Neuromuscular Centre workshops [72]. All patients were required to have received a muscle biopsy to verify diagnosis. A total of four centres participated in this study (Berlin (Germany), Duesseldorf (Germany), Lyon (France) and Paris (France). Patients were recruited from 2008 until 2019. No epidemiological or clinical differences were detected between centres (data not shown). Clinical and demographic data is in line with previous reports on ASyS patients. In addition to the ASyS cohort, three control groups were included as follows:

**Table 1:** Clinical characteristics of investigated patients and controls

		Jo-1 (*n* = 42)	PL-7 (*n* = 18)	PL-12 (*n* = 20)	Disease control (*n* = 40)	Non-disease control (*n* = 20)	Healthy control (*n* = 40)
Mean age at biopsy [y]		45 ± 14	55 ± 12	51 ± 18	52 ± 21	45 ± 9	49 ± 5
Biological gender	♀ / ♂	62% / 38%	50% / 50%	55% / 45%	65% / 35%	50% / 50%	50% / 50%
Additional antibodies*	Yes	**50%**	16%	**50%**	**65%**	0%	0%
	No	33%	**56%**	40%	20%	**100%**	**100%**
	Unknown	17%	28%	20%	15%	0%	0%
Creatine kinase	Normal	12%	11%	5%	25%	50%	**100**
(CK)	< Tenfold	26%	**50%**	**50%**	**20%**	**17%**	**–**
	> Tenfold	2%	22%	5%	10%	–	–
	> 30-fold	**40%**	–	20%	12%	–	–
	Unknown	19%	17%	20%	32%	33	–
C-reactive protein	Normal	**48%**	**44%**	**35%**	**25%**	**67%**	**67%**
(CRP)	< Tenfold	**19%**	**11%**	**30%**	**30%**	**–**	**–**
	> Tenfold	15%	11%	25%	15%	**–**	**–**
	Unknown	19%	33%	10%	30%	33%	33%
Onset of symptoms	< 1 year	26%	28%	50%	17%	33%	33%
	> 1 year	5%	22	–	26%	–	–
	> 5 years	–	–	–	–	17%	17%
	Unknown	69%	50%	50%	16%	50%	50%
Symptoms (yes/no/n.d)	Muscle symptoms						
	Pain	**81%** / 14% / 5%	**56%** / 33% / 11%	**80%** / 20% / 20%	**58%** / 29% / 13%	67%***/ – / 33%	–
	Weakness	**81%** / 10% / 10%	**50%** / 39% / 11%	**65%** / 30% / 5%	**81%** / 11% / 18%	67%***/ – / 33%	–
	Fatigue	**55%** / 29% / 17%	**50%** / 39% / 11%	**50%** / 40% / 5%	**71%** / 18% / 11%	– / – / **100%**	– / **100%** / –
	Lung involvement	**64%** / 24% / 12%	**56%** / 33% / 11%	**60%** / 35% / 5%	**42%** / 31% / 27%	– / – / **100%**	– / **100%** / –
	Skin involvement	**52%** / 38% / 10%	39% / **50%** / 11%	35% / **60%** / 5%	**58%** / 23% / 19%	– / – / **100%**	– / **100%** / –
	Raynaud	21% / **50%** / 29%	28% / **61%** / 11%	25% / **60%** / 15%	5% / **80%** / 15%	17% / – / **83%**	– / **100%** / –
	Cardiomyopathy	29% / **52%** / 19%	28% / **61%** / 11%	20% / **50%** / 5%	7% / **71%** / 18%	– / – / **100%**	– / **100%** / –
	Arthritis	**64%** / 24% / 12%	28% / **56%** / 17%	30% / **65%** / 5%	13% / **57%** / 30%	– / – / **100%**	– / **100%** / –
	Fever	37% / 33% / **52%**	17% / **50%** / 33%	30% / **40%** / 30%	– / 18% / **82%**	– / – / **100%**	– / **100%** / –
	Dysphagia	14% / **60%** / 26%	17% / **72%** / 11%	15% / **80%** / 5%	35% / **42%** / 23%	– / – / **100%**	– / **100%** / –
	Weight loss	12% / **52%** / 36%	22% / **56%** / 22%	30% / **55%** / 15%	7% / 25% / **69%**	– / – / **100%**	– / **100%** / –
	Neoplasm	– / **83%** / 17%	17% / **72%** / 11%	25% / **65%** / 20%	31% / **62%** / 7%	17% / – / **83%**	– / **100%** / –
Therapy	Corticosteroids	52%	28%	45%	68%	0	0
	Mean dose, mg (SD)	36.8 (25.5)	11.75 (18.9)	45 (37.7)	28.2 (19.1)	0	0
	DMARDs						
	– Naïve	**59%**	**68%**	**75%**	**52%**	0%	0%
	– Azathioprine	5%	11%	0%	11%	0%	0%
	– MMF	5%	0%	10%	0%	0%	0%
	– MTX	17%	5%	5%	25%	0%	0%
	– Cyclophosphamide	0%	11%	5%	0%	0%	0%
	– Other	14%	5%	5%	12%	0%	0%

^*^Additional antibodies included: anti-neutrophil cytoplasmic^+^ (ANCA), anti-ALAT^+^, anti-ASAT^+^, anti-CCP^+^, anti-NSMAF^+^, anti-Ro52^+^, anti-Ro60^+^, anti-SS-A^+^, anti-TPO

^**^One NDC had one measurement with a 2.8-fold increase; however, all others were normal

^***^Symptoms were subjective; in muscle biopsies no irregularities were found and no immune reactions and no signs of inflammation. CK levels were normal and no MSA or MAA were detected

- Healthy controls (HC, *n* = 40): The HC group were healthy individuals without known diseases. HC were used as control group for analysis of the peripheral immune response in ASyS. HC were sex- and age-matched to ASyS patients.
- Diseased controls (DC, *n* = 40): The DC group consisted of patients diagnosed with IIM other than ASyS (IMNM = 9, DM = 21, IBM = 10). The diagnosis was made according to current criteria [42]. DC patients were required to have no antibodies associated with ASyS. The DC cohort served to account for treatment bias. DCs were used for all analyses except for proteomics as described below.
- Non-diseased controls (NDC, = 20): NDC were patients that underwent muscle biopsy for diagnostic purposes but without any indication of inflammatory muscle disease, e.g., they had suffered from myalgia, but objective muscle weakness and morphological abnormalities on skeletal muscle biopsy were absent. CK levels were normal and no signs of systemic inflammation and no myositis-specific antibodies (MSA) or myositis-associated antibodies (MAA) were detectable. NDC were used as the control group for the analysis of changes to skeletal muscle in ASyS. NDC were sex- and age-matched to ASyS patients.

Overall, this study included 80 ASyS, 40 HC, 40 DC and 20 NDC. The individual number of patients is given in each experimental section. Informed consent was obtained from all patients and ethical approval was granted by the Charité ethics committee (EA2/163/17), as well as the local ethical committees in Düsseldorf and Essen (Düsseldorf: AZ 2021–1417, Essen: 19–9011-BO).

### Skeletal muscle specimens

In this study, we analyzed biopsied skeletal muscle, peripheral blood mononuclear cells and serum derived from ASyS patients and NDCs. All skeletal muscle specimens had been cryopreserved at  – 80 °C prior to analysis.

Availability and quality of material led to inclusion/exclusion of individual patients, depending on the analysis.

### Morphological analysis

All stains were performed on 8 µm cryostat sections, according to standard procedures. Immunohistochemical and double immunofluorescence reactions were carried out as described previously [57]. We used irrelevant antibody stains (either mouse/rabbit monoclonal/ polyclonal isotype controls) as negative controls, as well as omission of the primary antibody. The following antibodies were used for staining procedures:

APRIL (Abcam, polyclonal, 1:100), BAFF (Abcam, clone Buffy 2, 1:100), C5b-9 (DAKO, clone aE11, 1:200), CD4 (Zytomed, clone SP35, 1:100), CD8 (DAKO, clone 144B, 1:100), CD20 (DAKO, clone L26, 1:200), CD68 (DAKO, clone EBM1, 1:100), CD90 (Dianova, clone AS02, 1:100), CD138 (DAKO, clone MI15, 1:30), CD169/Siglec1 (Millipore, clone 5F1.1, 1:50), CXCL12 (R&D Systems SDF-1 MAb79018, 1:100), CXCL13 (R&D Systems, polyclonal, 1:100), CXCR4 (Abcam clone UMB2, 1:50), MHC-cl. I (DAKO, clone W6/32, 1:1000), MHC-cl. II (DAKO, clone CR3/43, 1:100), MyHC dev (Novocastra, clone NB-MHCd, 1:5), and MyHC neo (Novocastra, clone NB-MHCn, 1:20), PD1 (Abcam, clone NAT105, 1:100). Secondary antibodies for peroxidase (POD) staining: POD Goat Anti-Mouse or POD Goat Anti-Rabbit (Dianova, 1:100). Secondary antibodies for immunofluorescence: in general, AF488 combined with Cy3, AF568 and labeling of nuclei through DAPI containing liquid mounting medium. Antibodies: Goat Anti-Rabbit Alexa Fluor® 488, Goat Anti-Mouse Alexa Fluor® 488, Goat Anti-Mouse AF568, Goat Anti-Rabbit Cy3, or Goat anti-mouse Cy3 (all Dianova, 1:100).

For electron microscopy (EM), diagnostic muscle samples were processed as previously described [22]. Entire ultrathin sections with the near-absence of limiting artifacts (large-scale digitization samples; LDS) of resin blocks were prepared and digitized with a Zeiss Gemini 300 field-emission scanning electron microscope (Zeiss, Oberkochen, Germany), equipped with a scanning transmission electron microscopy detector (STEM) and Atlas 5 software (Fibics) with a scanning transmission electron microscopy detector (STEM) [22].

### Scores and cell counts

For further evaluation, a semi-quantitative score, as established previously [10], was used to rate the biopsies. The biopsies were blinded for scoring, with autoantibody status, age, sex or any other parameter not possible to identify from the label. The scoring was performed in randomly distributed 10 high power fields (HPF, based on the microscope used and the respective oculars ≙ 0.16 mm^2^), by four myopathologists (WS, HHG, CP, BP) leading to a mean overall severity score (1 = minimally affected and 10 = strongly affected). For this we rated the following multiple features:

Atrophy, necrosis, and regeneration each: 0 = absent, 1 = low, 2 = medium, 3 = strong. Perifascicular fragmentation: 0 = absent, 1 = seldom, perimysial, 2 = distinct, perimysial, 3 = peri-/endomysial. Perifascicular fibrosis and capillary enlargement, each 0 = absent, 1 = present. Capillary dropout: 0 = absent, 1 = present, singular, 2 = present, only perifascicular, 3 = present in multiple areas, extensive in whole fascicle. Upregulation of MHC I and MHC II, 0 = no staining; 1 = single fibers; 2 = perifascicular fibers, 1–2 cell layer; 3 = extensively in whole fascicle, > 2 cell layer. C5b-9 deposits: 0 = no deposits on sarcolemma; 1 = minimal number of deposits on sarcolemma; 2 = intermediate number of deposits; 3 = high number of deposits in whole fascicle. Immune cells (CD68, CD8, CD20 and CD138) were counted in ten HPF, which were evaluated per biopsy and the mean provided a score according to: 0 = no cells; 1 = single cells (1–4); 2 = 5–20 cells; 3 = cluster and/or > 20 cells.

### Quantitative reverse transcription PCR (qRT-PCR)

Total RNA was extracted from muscle specimens using a described previously technique [57]. Briefly, cDNA was synthesized using the High-Capacity cDNA Archive Kit (Applied Biosystems, Foster City, CA). For qPCR reactions, 10–20 ng of cDNA were used, and for subsequent analysis the 7900HT Fast Real-Time PCR System (Applied Biosystems, Foster City, CA) was utilized with the following running conditions: 95 °C 0:20, 95 °C 0:01, 60 °C 0:20, 45 cycles (values above 40 cycles were defined as “not expressed”). All targeted transcripts were run as triplicates. For each of these runs, the reference gene *PGK1* has been included as internal control to normalize the relative expression of the targeted transcripts. The qPCR assay identification numbers, TaqMan® Gene Exp Assay from Life Technologies/ ThermoFisher are listed as follows: APRIL/*TNFS13* Hs00182565_m1, BAFF/*TNFSF13B* Hs00198106_m1, *CCR7* Hs01013469_m1, *CXCL12* Hs03676656_mH, *CXCR4* Hs00607978_s1, *CXCL13* Hs00757930_m1, *CXCR5* Hs00540548_s1, *IL6* Hs00985639_m1, *IFNG* Hs00989291_m1, *TNFA* Hs00174128_m1, *IL1B* Hs01555410_m1, *STAT1* Hs01013989_m1, *PGK1* Hs99999906_m1. The ΔCT of NDCs was subtracted from the ΔCT of ASyS patients’ muscles to determine the differences (ΔΔCT) and fold change (2^-ΔΔCT) of gene expression. Gene expression was illustrated by the log10 of fold change values compared to NDCs.

### Flow cytometry

Cryopreserved peripheral blood mononuclear cells (PBMCs) from ASyS patients and sex- and age-matched healthy controls were thawed and stained with distinct sets of fluorochrome-conjugated antibodies (Supplementary Table 1). For intracellular staining, cells were treated with Fixation/Permeabilization solution (eBiosciences) for 20 min, subsequently washed with Permeabilization buffer (eBiosciences), and finally incubated with antibodies directed against intracellular target molecules of interest.

To investigate the capacity to produce cytokines, PBMC were rested overnight in X-Vivo 15 (Lonza) and subsequently stimulated with LAC (leukocyte activation cocktail, PMA, Ionomycin, Brefeldin A, BD Biosciences) for 4 h (h) prior to extra-cellular staining for lineage markers and intracellular staining for cytokines (for staining panels see Supplementary Table 1).

Flow-cytometric data were analyzed using Kaluza 2.1 Analysis Software (Beckman Coulter). Immune cell subsets were defined by the gating strategies outlined in Supplementary Table 2.

### Bead array for soluble factors

Concentrations of soluble factors (APRIL, BAFF, CD40L, CXCL12, IL-4, IL-6, IL-10, IL-13, IL-21, IFNγ, TNFα) in the serum of patient samples were analyzed by a LEGENDplex™ Multiplex Assay with a custom panel as per the manufacturer’s instructions.

### Multiplex analysis of complement factors

A multiplex ELISA based on chemiluminescence was used according to the manufacturer’s recommendations (Quidel Corporation, San Diego, CA, USA) to systematically profile complement proteins in serum samples as described previously [21]. Cut-offs were defined according to the manufacturer’s instructions.

### Muscle preparation for proteomics analysis

Lysis of the muscle samples was performed by adding 200 µl of 50 mM Tris–HCl (pH 7.8) buffer containing 5% SDS, and cOmplete ULTRA protease inhibitor (Roche). Samples were placed into the Bioruptor® (Diagenode) for 10 min (min) (30 s on, 30 s off, 10 cycles) at 4 °C. To ensure complete lysis, an additional sonication step with an ultra-sonic probe (30 s, 1 s/1 s, amplitude 40%) followed by centrifugation at 4 °C and 20,000 g for 15 min was conducted. Protein concentration was determined by BCA assay according to the manufacturer’s protocol. Reduction of disulfide bonds was done by addition of 10 mM TCEP at 37 °C for 30 min, and free sulfhydryl bonds were alkylated with 15 mM IAA at room temperature (RT) in the dark for 30 min. In total, 100 µg protein of each sample was taken for proteolysis using the S-Trap protocol (Protifi) and using a trypsin to protein ratio of 1:20. Digestion was conducted for 2 h at 37 °C. Proteolysis was stopped using FA to acidify the sample (pH < 3.0).

Complete digestion was checked for all proteolytic samples after desalting by using monolithic column separation (PepSwift monolithic PS-DVB PL-CAP200-PM, Dionex) on an inert Ultimate 3000 HPLC (Dionex, Germering, Germany) by direct injection of 1 μg sample. A binary gradient (solvent A: 0.1% TFA, solvent B: 0.08% TFA, 84% ACN) ranging from 5 to 12% B in 5 min and then from 12 to 50% B in 15 min at a flow rate of 2.2 μL/min and at 60 °C was applied. UV traces were acquired at 214 nm [15].

### Data independent acquisition liquid chromatography mass-spectrometry/mass-spectometry analysis (DIA LS-MS/MS)

All samples were analyzed using an UltiMate 3000 RSLC nano UHPLC setup coupled to a QExactive HF mass spectrometer by loading a total amount of 1 µg peptide per sample. The samples were first transferred to a 75 µm × 2 cm, 100 Å, C18 pre column with a flow rate of 10 µl/min for 20 min followed by a separation on the 75 µm × 50 cm, 100 Å, C18 main column with a flow rate of 250 nl/min and a linear gradient consisting of solution A (99.9% water, 0.1% formic acid) and solution B (84% acetonitrile, 15.9% water, 0.1% formic acid), where the pure gradient length was 120 min (3–45% solution B). The gradient was set up as follows: 3% solution B for 20 min, 3–35% for 120 min, followed by 3 washing steps each ranging to 95% buffer B for 3 min. After the last washing step, the instrument was allowed to equilibrate for 20 min. The acquisition of MS data was conducted in DIA (data independent acquisition) mode using a spectral library built in-house. An appropriate amount of iRT standard (Biognosys) was added to each sample analyzed. Full MS scans were acquired from 300 to 1100 m/z at a resolution of 60,000 (Orbitrap) using the polysiloxane ion at 445.12002 m/z as lock mass. The automatic gain control (AGC) was set to 3E6 and the maximum injection time to 20 ms. Full MS scans were followed by 23 DIA windows, each covering a range of 28 m/z with 1 m/z overlap, starting at 400 m/z, acquired at a resolution 30,000 (Orbitrap) with an AGC set to 3E6 and nCE of 27 (CID).

### Analysis of DIA data

Samples acquired with nano-LC–MS/MS in DIA mode were analyzed by introducing the data to the Spectronaut software (Biognosys) and analyzed with a library-based search. As the library, spectral library built in-house was used. Search and extraction settings were kept as standards (BGS Factory settings). As proteome background the human proteome data were selected from UniProt (www.uniprot.org) containing 20,374 entries. For reliable label-free quantification, only proteins identified with ≥ 2 unique peptides were considered for further analysis. Normalized relative intensities were obtained by Spectronaut and averages were calculated for each protein to determine the ratios between patient muscle samples and their respective controls. To investigate interactive networks, protein–protein interactions were visualized using STRING v11.5 (string-db.org).

### Statistical analysis

Since this is an exploratory and descriptive study, sample sizes are not based on a priori power calculation, but based on previous studies.

Data are presented as the median ± IQR unless otherwise indicated. Differences between two groups were examined by the Mann–Whitney *U* test. The Kruskal–Wallis test followed by the Bonferroni-Dunn correction for multiple comparisons was used to assess differences between multiple groups (*n* > 2). One-way analysis was performed using the Kruskal–Wallis one-way analysis of variance with Dunn’s multiple comparison test. Volcano plots were constructed by plotting log_2_ values of the relative difference between the median against the  – log_10_ *p*-values. Cut-off values were applied as indicated. Gene Set Enrichment Analysis (GSEA) was performed for proteomics data using the R package WebGestaltR (v0.4.4 [40]). The level of significance was set at *p* < 0.05. GraphPad Prism 8.4.3 software (GraphPad Software, Inc., La Jolla, CA, USA) was used for statistical analysis.

## Results

### Clinical description of anti-Jo-1, -PL-7 and -PL-12 autoantibody positive ASyS patients

80 patients with ASyS were included in this study. In 52.5% (*n* = 42) of cases, anti-Jo-1-autoantibodies were detected, followed by 22.5% (*n* = 18) anti-PL-7- and 25% (*n* = 20) anti-PL-12-autoantibodies. To elucidate potential clinical differences between serological subgroups, we analyzed clinical data according to the associated autoantibody status.

62% (26 patients) of anti-Jo-1^+^ and 55% (11 patients) of anti-PL-12^+^ patients were female, while in anti-PL-7^+^ patients there was an equal proportion of each gender (50:50). The mean age at time of biopsy did not differ significantly between the subgroups (Jo-1^+^ 45y ± 14y, PL-7^+^ 55y ± 12y, PL-12^+^ 51y ± 18y). CK levels varied among patients, with higher levels in anti-Jo1^+^ patients when compared to -PL-7^+^ and -PL-12^+^ patients (40% > tenfold vs. 20% / 25% > tenfold).

The presence of myalgia was more prevalent in anti-Jo-1^+^ (81%) and -PL-12^+^ (80%), when compared to anti-PL-7^+^ patients (56%). Anti-PL-7^+^ and -PL-12^+^ were associated with less skin involvement (PL-7 40% vs. PL-12 35% vs. Jo-1 52%) and arthritis (PL-7 28% vs. PL-12 30% vs. Jo-1 64%) compared to Jo-1^+^ patients. Additional symptoms comprised of fever, weight loss or heart involvement in a subset of patients. No neoplasms were detected the Jo-1^+^ patients, whereas neoplasms were found in 17–27% of participants in the other groups. Across all subgroups, patients were most often treatment-naïve for standard immunosuppressants. Approximately half of anti-Jo-1^+^ and anti-PL-12^+^ patients were treated with corticosteroids with a mean dose of 36.8 (25.5) mg and 45.0 (37.7) mg, respectively. 28% of anti-PL-7^+^ patients received corticosteroids with a mean dose of 11.75 (18.9) mg. None of the patients received B cell-depleting therapies. Further, we included a group of NDC (*n* = 20). This group was defined as controls that received muscle biopsy without indication of inflammatory muscle disease as described above. To account for treatment as a confounding factor, e.g., corticosteroids, we also included DC patients (*n* = 40). This group was recruited from non-specified myositis and DM patients who had evidence of inflammatory muscle disease. Importantly, this group was comparable in terms of treatment as 68% of patients received glucocorticoids with a mean dosage of 28.2 (19.1) mg. In this group, MSA other than anti-synthetase antibodies were detected, e.g., Mi-2, NXP2 or TIF1-γ. Clinical characteristics are given in Table 1.

Overall, clinical signs and symptoms of our cohort recapitulate what has been previously reported in the literature [30], which demonstrates that this cohort is a representative group of patients with ASyS-associated myositis.

### Blood analysis of cellular and soluble compartment reveals pronounced B cell related immune responses in ASyS

**Fig. 1:**
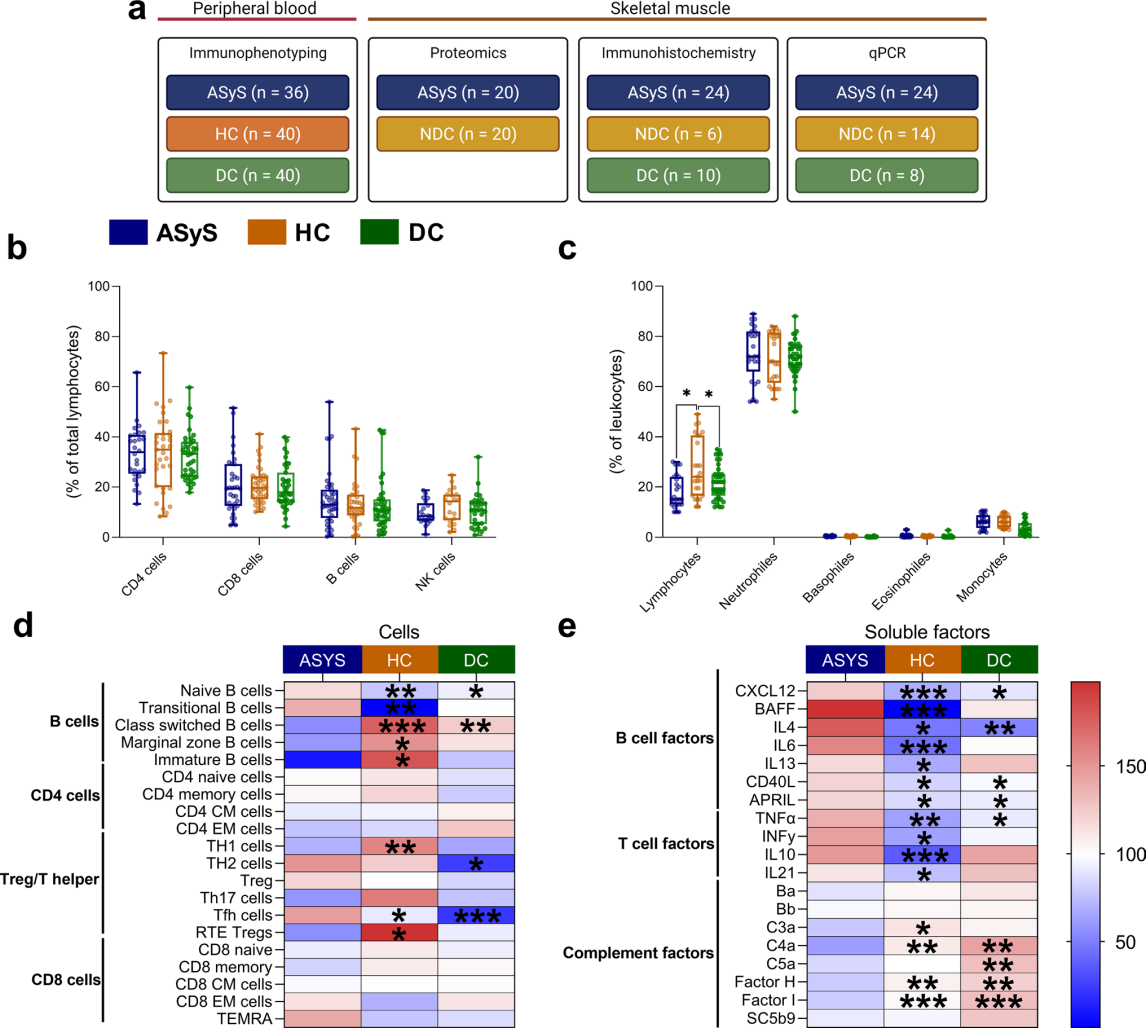
Immune cell profiling of the peripheral blood from ASyS patients demonstrates predominant alterations in the B cell compartment. Changes to peripheral immune cell subsets analyzed by flow cytometry for the leukocyte compartment (**a**) and the lymphocyte compartment (**b**) of ASyS (*n* = 36), and HC (*n* = 40) and DC (*n* = 40). In-depth analysis of the cellular immune compartment was performed by flow cytometry (**c**). Relative cell numbers were compared between ASyS, HC and DC for the corresponding immune cell compartments. Analysis of soluble factors was performed by bead array and multiplex immunoassay (**d**). Serum levels of soluble factors were compared between ASyS, NDC and DC as indicated. The heatmap indicates the normalized group mean as color code. Significance is indicated for the comparison ASyS vs. HC and for ASyS vs. DC in each row, respectively. Also see suppl. Figure 1 for detailed comparison of the peripheral immune response. Differences between groups were analyzed using the Kruskal–Wallis test followed by the Bonferroni-Dunn correction for multiple comparisons. *p < 0.05. *ASyS*  Anti-synthetase syndrome, *CM *central memory, *EM *effector memory, *NDC *non-diseased control, *RTE *recent thymic emigrates

To characterize the immunological phenotype of ASyS, different immune cell subsets of peripheral blood mononuclear cells and soluble inflammatory factors in the serum were investigated. For analysis of the peripheral immune response, we analyzed ASyS (*n* = 36), HC (*n* = 40) and DC (*n* = 40). We performed analysis of PBMCs to obtain flow cytometric data on immune cell composition. For soluble factors, we employed both bead arrays and multiplex analysis to investigate serum levels. At the cellular level, a subtle reduction in lymphocytes counts with similar granulocyte and monocyte levels were detected in ASyS and DC compared to HCs (Fig. 1a). In the lymphocyte compartment, frequencies of CD4^+^, CD8^+^ T, B and NK cells were comparable across groups (Fig. 1b). In contrast, further differentiation of subset composition revealed pronounced alterations in the B cell compartment in ASyS compared to HCs and DCs. Naïve B cell populations were significantly expanded, whereas mature B cell subsets (class-switched, marginal zone and IgM only memory B cells) were decreased in ASyS (Fig. 1c, Supplementary Fig. 1a). This effect was less pronounced compared to DCs. Plasma cells were not detected in the blood of ASyS patients or control groups. In the CD4^+^ T cell compartment, cellular subsets including naïve and memory CD4^+^ T cells were comparable across groups (Fig. 1c, Supplementary Fig. 1b, c). Further, reduced proportions of T helper 1 (T_H_1) and recent thymic emigrant regulatory T cells (RTE-Treg) were detected in ASyS compared to HCs, whereas Tfh cells were expanded compared to DCs and recent thymic emigrant regulatory T cells (RTE-Treg) were decreased compared to HCs. CD8^+^ T and NK cell subsets demonstrated a similar distribution across groups (Fig. 1c, Supplementary Fig. 1d, e). In accordance with the marked alterations detected in the B cell compartment, increased serum concentrations of several soluble factors related to B cell activation, proliferation and maturation (APRIL, BAFF, IL-4, IL-6, IL-13, IL-21, sCD40L [60]) were detected, as well as evidence of CXCL12-mediated B cell migration [60, 75] (Fig. 1d, Supplementary Fig. 1f) in ASyS patients. This change was less pronounced when compared to DC patients. As antibodies are supposed to be essentially involved in the pathogenesis of ASyS and essential for classical complement activation, serum concentrations of different complement factors were evaluated. In comparison to HCs and DCs, higher levels of C4a, C3a and the regulatory Factor H and Factor I were observed, whereas Ba, Bb, C5a and soluble C5b-9 levels were similar between groups (Fig. 1d, Supplementary Fig. 1 g, Supplementary Table 3). Regarding T cell-related cytokines, increased concentrations of IL-10, IL-21, IFN-γ and TNF-α in the serum of AsyS patients could be detected compared to HCs but not DCs (Fig. 1d, Supplementary Fig. 1 h). Taken together, we observed marked alterations of the peripheral immune response in ASyS. At the cellular level, a pronounced B cell response was evident, with both soluble B and T cell factors aberrant in ASyS. In addition, multiple complement factors were elevated in ASyS compared to HCs and DCs. To assess the potential influence of glucocorticoids on the peripheral immune response, we divided our ASyS cohort according to those who did (*n* = 17) or did not (*n* = 19) receive glucocorticoid treatment. Comparing these two groups, we observed only subtle effects consistent with the known effects of glucocorticoids on immune cell composition (Supplementary Fig. 2). Lymphocyte and monocyte numbers were decreased in glucocorticoid-treated patients, while the number of immature B cells was slightly increased.

### Blood analysis of cellular and soluble compartment demonstrates subtle differences in ASyS autoantibody subtypes

**Fig. 2:**
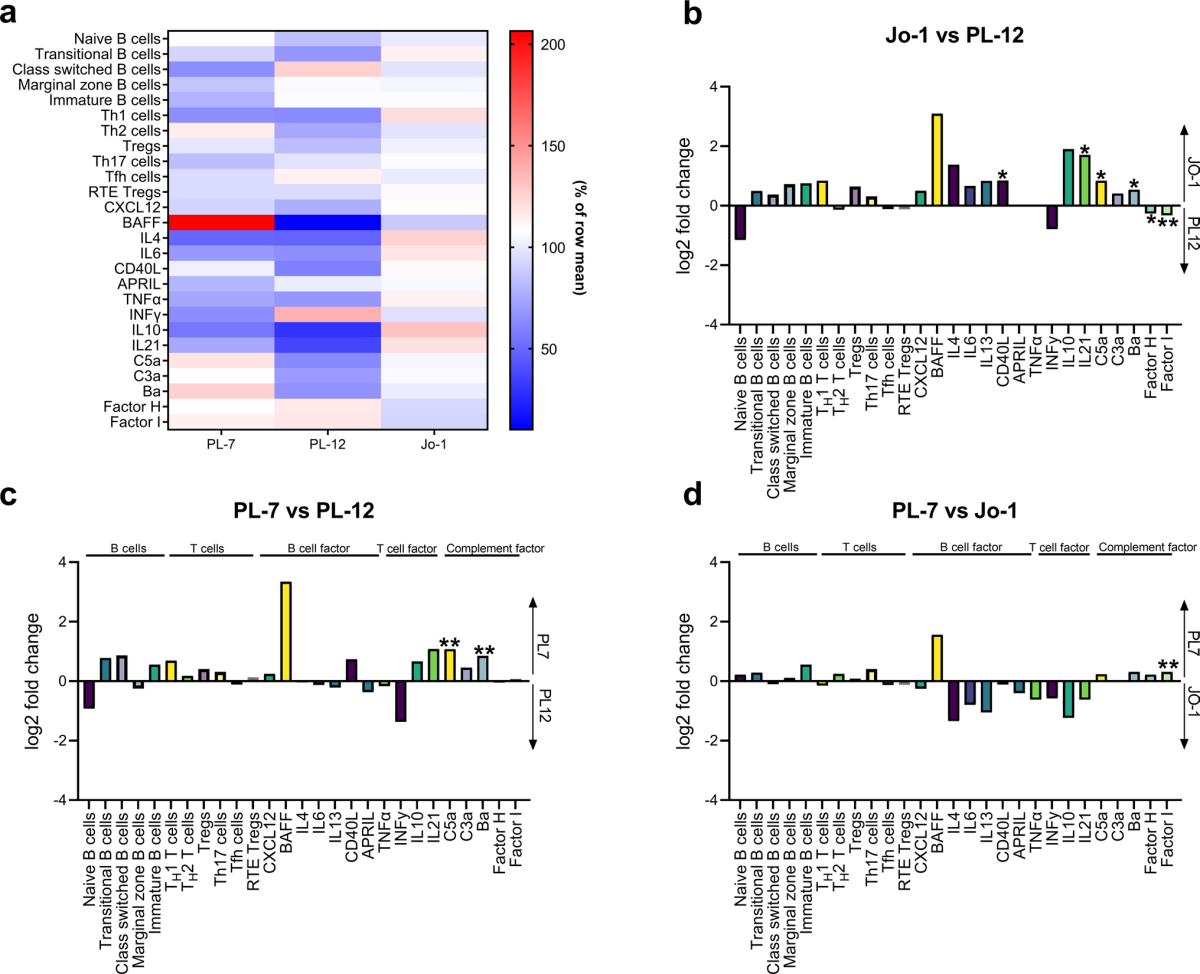
Immune profiles demonstrate subtle differences between serological ASyS subgroups. In-depth immune analyses as performed in Fig. 1 were compared for serological ASyS subgroups: anti-Jo-1^+^ (*n* = 22), PL-7^+^ (*n* = 8) and PL-12^+^ (*n* = 6). Heatmap displaying changes to cellular and soluble factors for serological subgroups (**a**). Data is displayed as median with normalization to the row mean. Two-sided comparison between subgroups is displayed as bar graph (**b**, **c**, **d**) with log_2_ fold change of the median on the y-axis. Significant differences between groups are indicated. Differences between groups were analyzed using the Kruskal–Wallis test followed by the Bonferroni-Dunn correction for multiple comparisons. The level of significance was set to *p* < 0.05. *p < 0.05, **p < 0.01. *CM* central memory, *EM* effector memory, *RTE* recent thymic emigrates

Next, we aimed to dissect patterns of immunological alterations in relation to serological subgroups. Therefore, we investigated alterations in the parameters stated above that distinguished ASyS from NDCs. In many aspects, the patterns were similar among the three ASyS subgroups. However, blood-derived parameters were the most similar between anti-Jo1 and anti-PL7 ASyS (Fig. 2a). At the cellular level, all subgroups were highly similar (Fig. 2a–d). In contrast, of the soluble immune parameters, the observed patterns in anti-PL-7 and anti-Jo-1 ASyS were indicative of stronger B cell responses and complement activation compared to anti-PL-12. However, the differences reached statistical significance only for single parameters (Fig. 2b–d). In conclusion, the different serological subgroups demonstrated only subtle differences in their cellular and non-cellular blood immune phenotype.

### Skeletal muscle pathology is similar among autoantibody subtypes in ASyS-associated myositis

**Fig. 3:**
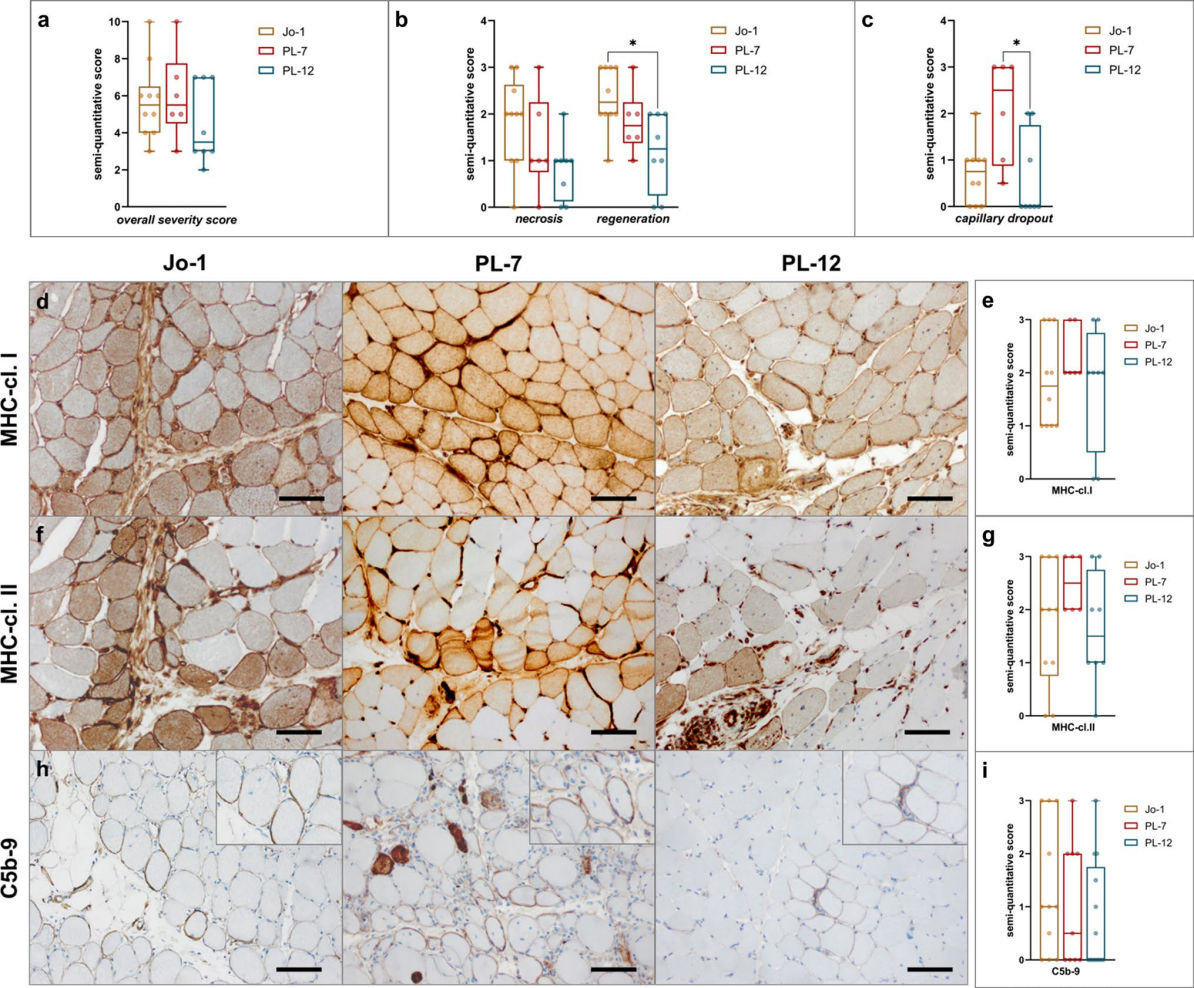
Muscle morphological features of ASyS were similar between serological ASyS subgroups. Analysis of skeletal muscle biopsies by immunohistochemistry (**a**, **b**, **c**, **e**, **g**, **i**). Muscle morphology was quantified and compared between subgroups by semi-quantitative score. Muscle biopsies from anti-Jo-1^+^ (*n* = 10), PL-7^+^ (*n* = 6) and PL-12^+^ (*n* = 8) patients were analyzed. Representative muscle biopsies are displayed (**d**) for C5b-9, MHC-cl. I and MHC-cl. II for serological subgroups. Scale bar 100 µm. Differences between groups were analyzed using the Kruskal–Wallis test followed by the Bonferroni-Dunn correction for multiple comparisons. The level of significance was set to *p* < 0.05. *p < 0.05. *cl.* = *class*

Aside from clinical and blood-derived data, we quantified morphological features such as atrophy, necrosis, regeneration, sarcolemmal MHC expression and cell infiltration by assessment of different immune histological stains. Regarding the overall severity score, there were no significant differences among subgroups (Fig. 3a). However, upon further analysis, higher level of necrosis and regeneration could be observed in Jo1^+^ patient’s muscles, without reaching statistical significance (Fig. 3b). Loss of capillaries was pronounced in the muscles of anti-PL-7^+^ patients (Fig. 3c).

Up-regulation of MHC-I was strong and found in all patients with ASyS (Fig. 3d, e), as was expression of MHC-II, pronounced in the perifascicular areas, clearly separating AsyS patients from those with other forms of myositis and indicating type I and type II interferon responses within the muscle. Only single anti-PL-12^+^ patients were negative for MHC-II (Fig. 3f, g). Consistent with this, we detected significantly upregulated gene expression of *IFNG*, *TNFA, IL6, IL1B* and *STAT1,* which indicate a prominent type II interferon response in the skeletal muscles of all three subgroups (Supplementary Fig. 3). In accordance with this, we detected significantly increased concentrations of IFNγ and TNFα in the sera of ASyS patients compared to those of NDC (Supplementary Fig. 1 h).

Sarcolemmal deposition of the C5b-9 complex, indicating activation of terminal complement, was found in around 50% of ASyS patients, whereby amount of deposition varied between and within subgroups (Fig. 3h, i). Jo-1^+^ patients had higher scores than PL-7^+^ or PL-12^+^ patients, but this did not reach statistical significance.

In summary, the general pathology of patients’ biopsied muscles with ASyS-associated myositis was similar between antibody subgroups [24, 47, 68].

### Proteomic analysis of skeletal muscle indicates increased antigen presentation and processing and disturbed muscle homeostasis in ASyS patients

**Fig. 4:**
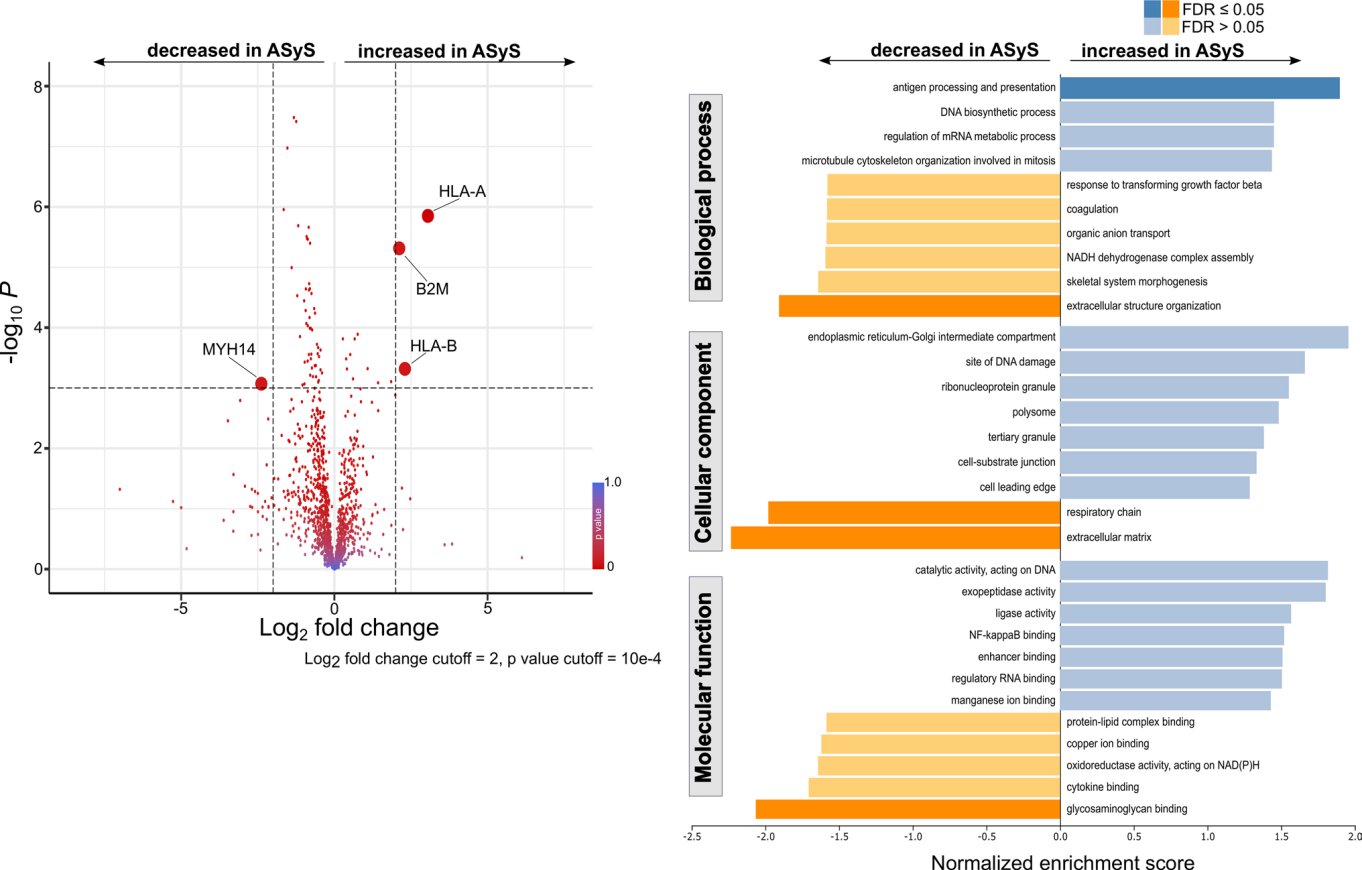
Proteomic profiling identifies for enhanced antigen-processing and presentation in skeletal muscle of ASyS patients. Analysis by proteomic profiling of muscle biopsies from ASyS patients (*n* = 20) and NDC (*n* = 20) displayed as volcano plot (**a**). Volcano plots were constructed by calculating the log_2_ fold change of the median and the -log_10_ p-value. The log_2_ fold change and *p*-value cutoff are indicated. Differentially expressed proteins are bold. Functional pathways were assessed by gene set enrichment analysis for biological processes, cellular components and molecular functions (**b**). The significance level was set to the FDR-adjusted *p* < 0.05. Statistical analysis was performed using the Mann–Whitney test (unpaired comparisons). *ASyS* Anti-synthetase syndrome, *NDC*  non-diseased control, *B2M*  beta2-microglobulin, *FDR*  false discovery rate, *HLA*  human leukocyte antigen, *MYH14*  myosin-14

Aiming to further dissect the immunogenic milieu of skeletal muscle, proteomic profiling of muscle biopsies from NDCs and ASyS patients was applied. The comparison of proteomic signatures revealed a pattern of proteins elevated in ASyS responsible for antigen presentation and processing, particularly members constituting the MHC class I complex (Fig. 4a). Concurrently, myosin-14 (MYH14) was decreased in ASyS. Analysis of functional pathways demonstrated the immunogenic properties of skeletal muscle and further supported that proteins for antigen processing and presentation were significantly enriched in ASyS (Fig. 4b). Additionally, pathways for the cellular components and molecular functions were identified. A number of protein pathways responsible for the maintenance of skeletal muscle homeostasis were decreased in ASyS, including the respiratory chain, extracellular matrix and glycosaminoglycan binding. In-depth analysis of proteins associated with antigen presentation and processing using STRING analysis revealed that in addition to MHC class I, antigen processing is driven by the 20 s proteasome and the AP2 adaptor complex in ASyS (Supplementary Fig. 4a). Expression of calreticulin (CALR) and Protein Disulfide Isomerase Family A Member 3 (PDIA3), belonging to the MHC class I, were corroborated on protein level using immunohistochemistry (Supplementary Fig. 4b, c). Both proteins contribute to formation of MHC class I [27, 59]. In line with our previous analysis of ASyS autoantibody subtypes, proteomic profiling of skeletal muscle revealed no meaningful differences between Jo-1^+^, PL-7^+^ or PL-12^+^ subtypes (Supplementary Fig. 5). Thus, proteomic analysis supports an antibody-mediated pathology in ASyS.

### ASyS-associated myositis is characterized by strong macrophage infiltration as well as B cell / plasma cell clusters

**Fig. 5:**
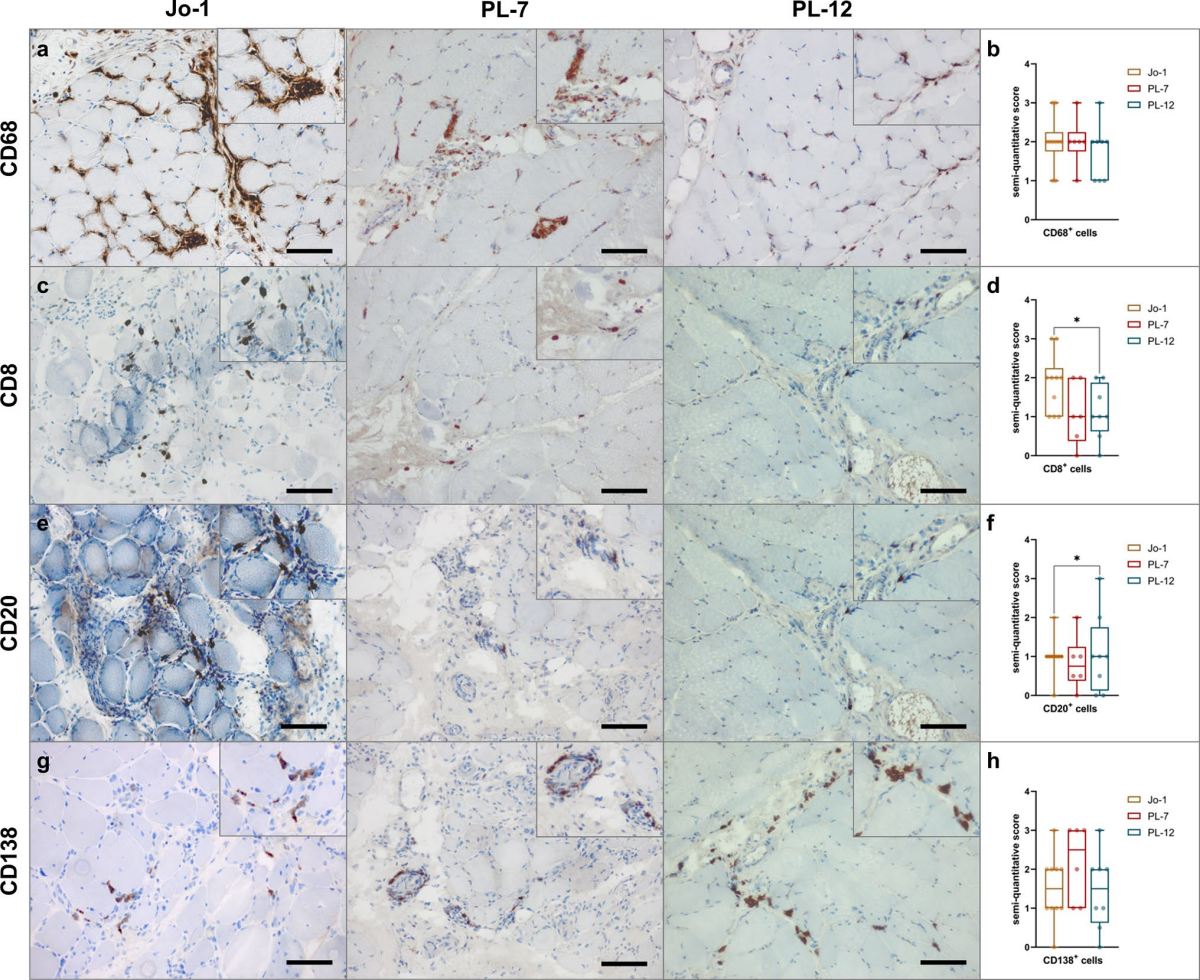
Macrophage-rich infiltrates with B cell/plasma cell clusters are characteristic for ASyS-associated myositis. Representative immunohistochemical stains of muscle biopsies from anti-Jo-1^+^ (*n* = 10), PL-7^+^ (*n* = 6) and PL-12^+^ (*n* = 8) patients for CD68, CD8, CD20 and CD138 (**a**). Scale bar 100 µm. Prominent invasion by CD68^+^ macrophages and T cells into the perimysium and the adjacent endomysial areas was observed. Muscle morphology was quantified and compared between subgroups by semi-quantitative score. Representative muscle biopsies are displayed (**d**) for C5b-9, MHC-cl. I and MHC-cl. II for serological subgroups. Scale bar 100 µm. Differences between groups were analyzed using the Kruskal–Wallis test followed by Bonferroni-Dunn correction for multiple comparisons. The level of significance was set to *p* < 0.05. *p < 0.05. *ASyS* Anti-synthetase syndrome, *NDC* non-diseased control

A distinct feature of ASyS is the specific infiltration of immune cells into the muscle tissue orchestrating the inflammatory response. Therefore, we analyzed the muscle-infiltrating immune cells and their location within the muscle by histology and semi-quantitative analyses. First, these analyses revealed a prominent invasion of the perimysium and the adjacent endomysial areas by CD68^+^CD169^+^ macrophages, with these being the most frequent invading immune cells (Fig. 5a, b). T cells were predominantly observed in the perimysial areas. CD8^+^ T cells were found most abundantly in anti-Jo-1^+^ patients (Fig. 5c, d). Of note, we identified an ample number of CD20^+^ B cells (Fig. 5e, f) and CD138^+^ plasma cells (Fig. 5g, h), again predominantly in the perimysium, extending into the endomysium, mostly in clusters, which are rare in other forms of myositis. Thus, cellular infiltrates were similar between the serological subgroups. In all subgroups, we found characteristic perimysial B cells/plasma cells.

### Skeletal muscle perimysium produces a specific immune micro milieu for B cells and plasma cells in ASyS

**Fig. 6:**
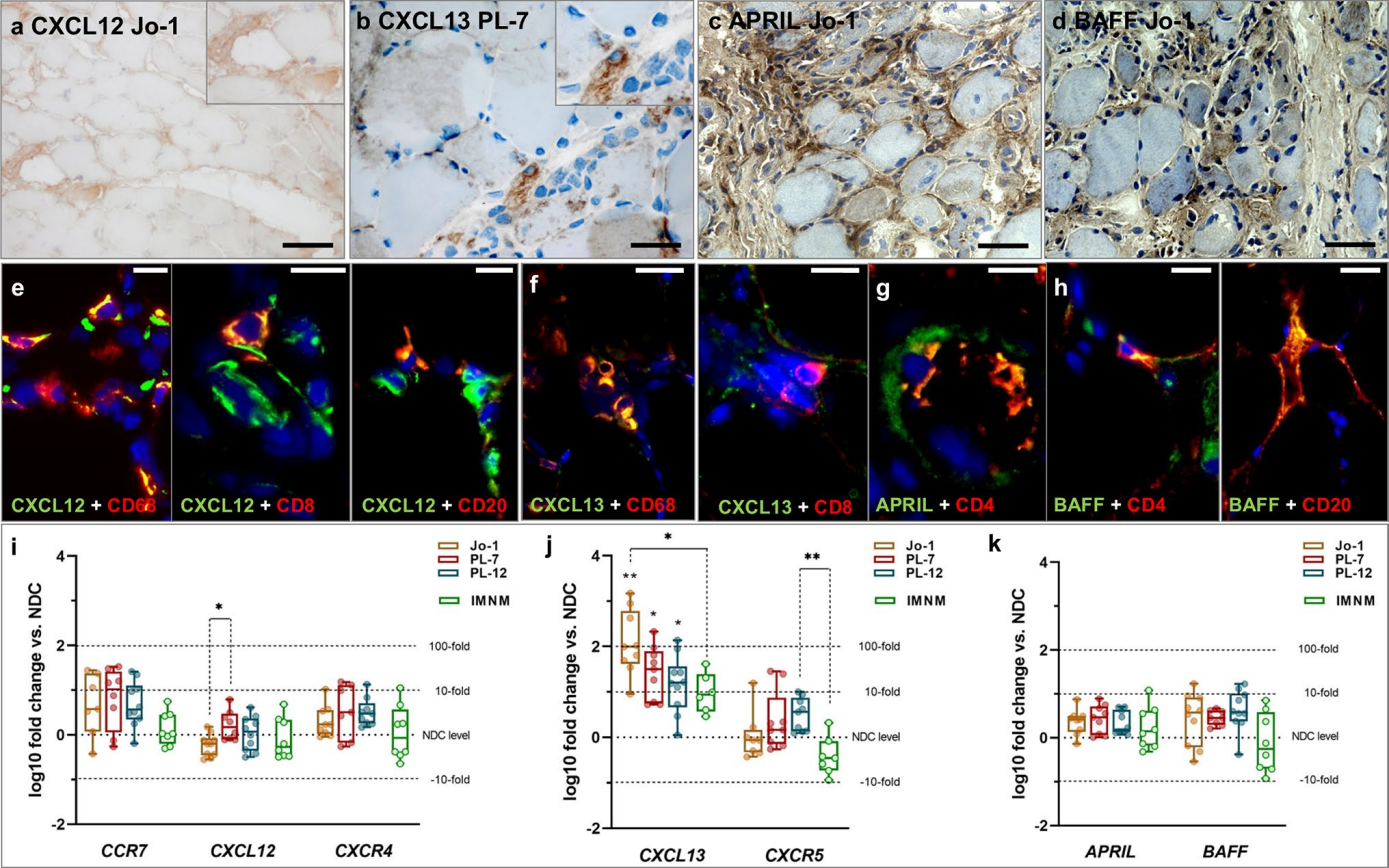
B cell homing and transcription factors constitute a pro-inflammatory micro milieu in ASyS patients’ skeletal muscle. Representative stains of muscle biopsies from ASyS patients with corresponding serological status. Monocytic cells in the inflammatory infiltrate of the skeletal muscles in ASyS express CXCL12 (**a**), CXCL13 (**b**) APRIL (**c**) and BAFF (**d**). Scale bar 100 µm. Immunohistological co-stains for immune cells with CXCL12 (**e**), CXCL13 (**f**), APRIL (**g**) and BAFF (**h**) are displayed as representative pictures, scale bar 25 µm. CXCL12 is co-expressed in CD68^+^ macrophages, CD8^+^ T cells and CD20^+^ B cells, while CXCL13 is co-expressed in CD68^+^ macrophages and CD8^+^ T cells. Additionally, CD4^+^ T cells express APRIL and BAFF, which are also found in CD20^+^ B cells. Analysis of gene expression of *CCR7*, *CXCL12* and *CXCR4* (i), *CXCL13* and *CXCL5* (**j**), as well as *APRIL* and *BAFF* (**k**) are displayed as box-plot indicating the log10 fold change of ASyS compared to NDC for corresponding serological groups, as well as in IMNM patients, which serve as the disease control (DC) group (**i**, **j**, **k**). Significant differences comparing ASyS or DC with NDC are indicated by the corresponding *p*-value above each plot. Significant differences between serological groups is indicated by the corresponding *p*-value connecting groups by dotted lines. The Kruskal–Wallis one-way analysis of variance with Dunn’s multiple comparison test was used. The level of significance was set to **p** < 0.05. *p < 0.05, **p < 0.01, ***p < 0.001. *ASyS*  Anti-synthetase syndrome, *DC*  disease control, *IMNM*  immune-mediated necrotizing myopathy, *NDC*  non-diseased control

Based on those specific B cell/plasma cell patterns in ASyS muscle, we next investigated the humoral immune response and the immune micro milieu in detail. Therefore, we analyzed the expression of chemoattractants involved in homing of certain B cell and plasma cell subtypes within the skeletal muscle biopsies. We observed that CXCL12 and the respective chemokine receptor CXCR4 were all expressed on monocytic cells in the inflammatory infiltrate of the skeletal muscles of all three ASyS subtypes (Fig. 6a, Supplementary Fig. 6a, b). CXCL12 co-expression was detected in CD68^+^ macrophages, CD4^+^ and CD8^+^ T cells and CD20^+^ B cells (Fig. 6d, e, Supplementary Fig. 6a), while CXCL13 was co-expressed by CD68^+^ macrophages, CD8^+^ T cells and CD20^+^ B cells (Fig. 6b, f, Supplementary Fig. 6c). Neither gene expression of *CCR7* nor *CXCR4* was elevated in ASyS or IMNM patients (an acute inflammatory myopathy serving as DC) compared to NDC, while *CXCL12* expression was elevated in PL-7^+^ patients when compared to Jo-1^+^ patients (Fig. 6i). In addition, expression of *CXCL13* was increased by more than 1.5-fold in all subgroups, reaching significance in all ASyS patients compared to NDC, as well as in Jo-1^+^ patients compared to DC (Fig. 6j). Expression of its receptor *CXCR5* was not increased in ASyS patients vs. NDC, but PL-12^+^ patients had significantly more *CXCR5* expression than DC (Fig. 6j). No significant up-regulation of *APRIL* and *BAFF* was detected in any of the groups (Fig. 6k), which are pivotal for survival and homing of plasma cells. However, strong expression of those factors was detectable instead at the protein level (Fig. 6c, d). BAFF expression was observed in CD20^+^ B cells, but not in CD138^+^ plasma cells (Fig. 6h, Supplementary Fig. 6e). However, expression of APRIL could not be detected in these cells (Fig. 6g, Supplementary Fig. 6d), but in macrophages and monocytes instead (e.g., CD4^+^ T cells, Supplementary Fig. 6d). Generally, those B cell / plasma cell factors were predominantly found in the perimysium extending into the endomysium. Additionally, corresponding stains were performed on skeletal muscle tissue of DC patients, demonstrating markers being expressed by the same immune cells. However, since quantification of, e.g., B cells revealed lower numbers in IMNM patients, the overall abundance of the respective chemokines/receptors was less relevant than in muscle tissue from ASyS patients (Supplementary Fig. 7).

In conclusion, the skeletal muscle of ASyS patients provides a specific micro milieu for B cell / plasma cell homing, survival and maturation.

### Endomysial AP positive fibroblasts, macrophages and muscle fibers form a plasma cell niche in ASyS patients

**Fig. 7:**
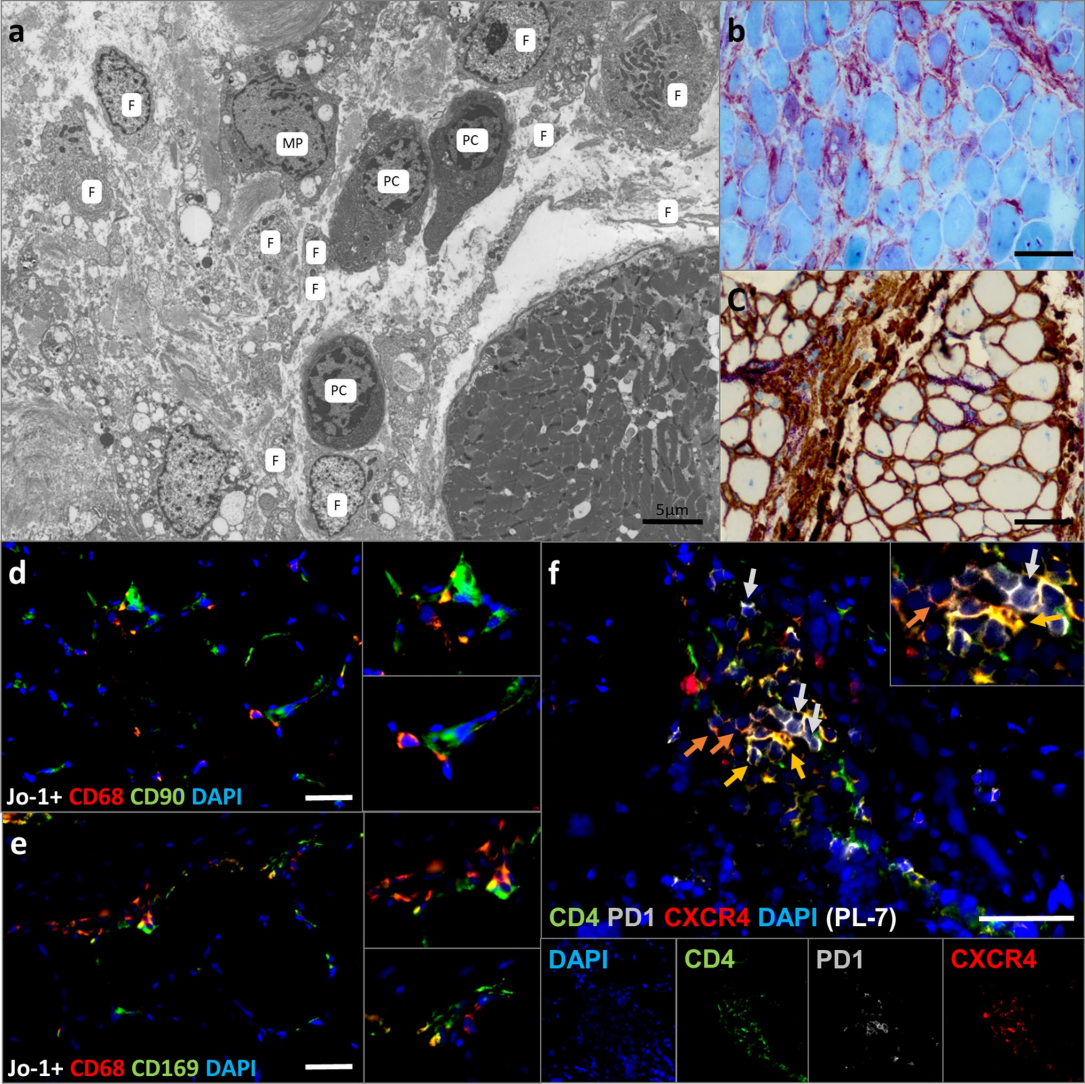
Electron microscopy dissects the plasma cell micro milieu in ASyS patients. Representative electron microscopy (EM) from the perimysial area of a muscle fascicle displays the composition of the immunological micro milieu with plasma cells (P), fibroblasts (F) and a putative macrophage (MP) (**a**). Scale bar 5 µm. Light microscopic imaging reveals AP-positive areas (red/purple) probably due to presence of activated CD90^+^ fibroblasts (brown) in muscle of ASyS patients (**b**, **c**). Scale bar 100 µm. Representative immunofluorescence microscopic images display CD90^+^ fibroblasts in close proximity to CD68^+^ macrophages (**d**). Individual macrophages also stain positive for CD169/Siglec1^+^ (e). Additionally, CD4^+^CXCR4^+^ T cells (yellow), as well as CD4^+^PD1^+^CXCR4^+^ Tfh cells (orange) are found in these areas (**f**). Scale bar **a** = 5 µm, **b**, **c** = 50 µm, **d**–**f** = 25 µm

We further characterized the cellular micro milieu in the skeletal muscle interstitial tissues using electron microscopy (EM) and histology. EM demonstrated plasma cells (PC) with typical eccentric nuclei and rough endoplasmic reticulum-rich cytoplasm, closely intermingled with fibroblast (F) processes and cell bodies. A larger cell with a slightly darker appearance, intracytoplasmic vacuoles and small cytoplasmic protrusions resembles a macrophage (MP) (Fig. 7a). The area shows strong alkaline phosphatase (AP) staining (Fig. 7b), whereby part of this reaction is due to AP-positive CD90^+^ fibroblasts (Fig. 7a, c). Fibroblasts are located in close proximity to macrophages (Fig. 7d), which are numerous and (partially) CD68 and CD169 (Siglec-1) double-positive (Fig. 7e). Additionally, various T cells were identified (Fig. 5c). Myofibers predominantly in the perifascicular areas were positive for MHC-I and -II (Fig. 3d, f), indicating an additional type II interferon response within the muscle, which is relevant in the context of plasmablast signaling. There was no specific endothelial capillary or arteriolar association with the plasma cells/plasmablasts. However, single CD4^+^CXCR4^+^ T cells and interestingly also CD4^+^PD1^+^CXCR4^+^ Tfh cells were also found in these areas [76, 77]. Local enrichment of Tfh supports the hypothesis of a supportive immunological niche for the plasma cells and activated B cells in perimysial areas.

## Discussion

In this study, the major three subgroups of ASyS-associated myositis, anti-PL-1 (Jo-1), -PL-7 and -PL-12 autoantibody-associated ASyS, demonstrated overlapping pathological features in one of their target organs, the skeletal muscle. Of note, we identified a highly specific immune phenotype in situ, characterized by presence of plasma cells/plasmablasts as well as Tfh clusters within the perimysium/perimysial areas of affected skeletal muscle and aberrant B cell pathology within the blood. Presence of those cells in the muscle was associated with a micro milieu that provides homing and survival factors, which are expressed by activated mesenchymal AP^+^ fibroblasts, associated lymphomonocytic cells and MHC-I^+^ and -II^+^ myofibers expressing type I and type II interferons mostly at the rim of the muscle fascicle. Our findings in peripheral blood support a strong type II interferon response. Muscle proteomics revealed enhanced antigen processing and presentation in ASyS patients. Thus, we hypothesize that the skeletal muscle provides sufficient elements and features qualifying as a survival micro milieu for memory plasma cells [35], and that these cells via secretion of the respective ARS autoantibodies may be crucially involved in chronicity of ASyS.

We and others have recently identified that the skeletal muscle pathology in ASyS is distinct from other types of idiopathic inflammatory myopathy [3]. This notion is based on myopathological and immune features including the presence of MHC-I and MHC-II on myofibers [51, 54]. MHC class I expression is evidenced on myofibers, showing an intriguing perifascicular pattern different from DM or IMNM patients [8, 63, 68]. MHC class I overexpression is further corroborated by changes to the muscle proteome of ASyS as compared to NDC. Further, MHC class I expression is not limited to ASyS, but has also been described across IIM entities [20]. Besides, we also observed a prominent perimysial inflammatory milieu with AP^+^ fibroblasts, presence of many Siglec-1^+^ macrophages, T cells as well as perifascicular necrotic myofibers, including decoration of non-necrotic myofibers in this area by C5b-9, and nuclear actin inclusions. In this study, immune phenotyping is expanded to the *in depth* description of foci of plasma cells within this specific perimysium providing homing (CXCL12, CXCL13) and survival factors (APRIL, BAFF) for memory plasma cells [35]. Of note, activities of factors such as CXCL12 are promiscuous and not limited to the B cell compartment. CXCL12 is also known to stimulate T cells and acts as chemotactic for lymphocytes [36]. Further, we describe upregulation of CXCL13, a central homing factor for B cells [7]. Levels of the CXCR5 receptor mediating CXCL13 signaling on B cells were unchanged compared to controls, potentially owing to the dynamic function and secretion of CXCL13 [18]. Further, we also describe clusters of Tfh cells, potentially providing support for germinal centers and B cell maturation. The latter are of particular interest given these cells role for B cell maturation and antibody function [19]. Tfh cells provide IL-21 and CD40L signaling required for B cell differentiation [19]. Most B cells are unable to successfully mature without sufficient Tfh cell interaction [38]. It is interesting to note that Tfh cells are implicated in antibody-mediated autoimmunity, i.e. Sjögren syndrome [70], however, their role in IIM remained largely elusive. A previous study reported increased levels of Tfh cells and IL-21 in peripheral blood of IIM patients compared to HC [76]. Concurrently, detection of Tfh clusters in the perimysial area of ASyS muscle in our study indicates Tfh/B-cell interaction as a pathogenic mechanism in IIM, particularily in ASyS. Consistent with a pathogenic role of B cell/plasma cell foci, a recent publication by Talim et al*.* [61] observed plasma cells in more than half of their biopsies in juvenile myositis. The authors identified a specific micro milieu with presence of IL-4 and type I as well as type II interferons. Analysis of the V(H) repertoire and clonal diversification of B cells in myositis (not associated to any autoantibodies) argued that B cells are actively proliferating and that oligoclonal populations occur in situ, whilst follicular dendritic cells (FDCs) as counterparts for antigen presentation were not found.

While in DM a highly specific type I interferon response is mainly present, the interferon response in ASyS patients is critically different from that in other forms of DM, involving a mixed type I and type II response [12, 13, 26, 55]. Here, we expand these data and provide evidence for MHC-II-positive myofibers as major contributors to IFNγ-driven immunity. We found strong MHC-II expression in all three subtypes at the mRNA and protein levels being a prerequisite for plasmablast maturation and differentiation into plasma cells [50]. The observed reduction of Th1 cells and increased TNFα and IFN-γ levels in the peripheral blood in ASyS patients further argue for a strong type II interferon response in ASyS.

Consistent with increased expression of antigen presentation molecules in histological and immunhistological stainings, our proteomic data revealed enhanced antigen processing and presentation in skeletal muscles of ASyS patients. Local antigen-driven humoral responses have been reported in myositis in general as IgG class switched plasma cells were detected in muscle biopsies [14]. However, this previous study observed only subtle expansion of B cells and plasma cells, which supports our observation that this cellular response is a signature of ASyS and in some cases of anti-Mi-2 associated DM [11]. Isolation of single plasma cells by laser microdissection in muscle affected by myositis identified a permissive environment including T cells and dendritic cells, as well as demonstrated that B-cell maturation had occurred. Interestingly, the authors concluded at that time, 2010, ‘*the clinical implications of B‐cell maturation and dendritic‐cell antigen presentation locally in muscle are uncertain’*. [62]

Most studies link specific local B cell and plasma cell immunity to the presence of so-called extranodal germinal centers within inflamed tissues. We also studied those secondary lymphoid organs extensively in DM and identified a unique immune phenotype [58]. In contrast, we could not detect extranodal germinal centers, but plasma cell foci in close proximity to fibroblasts and macrophages as observed by electron microscopy in all ASyS subgroups, most notably in the perimysial area and periymsium. Macrophages could be classified as expressing CD68^+^CD169^+^. These macrophages are primarily found in secondary lymphoid organs ensuring long-term antigen presentation to B cells [41]. A strong BCR signal is essential for the generation of memory plasma cells [65]. Furthermore, fibroblasts provide potential survival niches for long-lasting plasma cells [35]. Thus, our ultrastructural analyses further confirm a specific plasma cell niche in ASyS potentially influencing the pathophysiological reaction, since memory plasma cells might be able to chronically produce the disease-inducing ARS. Consistent with an antibody-mediated disorder, we found complement pathway activation in the muscle fibers (sarcolemmal) and blood of ASyS patients. Prominent capillary and sarcolemmal complement deposition can also be found in DM, while sarcolemmal-predominant complement is most often found in Mi-2 positive DM [71].

Consistent with previous reports, we observed an increase of immature B cell subsets in the peripheral blood, whereas memory B cell subsets were decreased in ASyS patients [24]. This might be related to enhanced migration into the muscle compartment, which is supported by the highly abundant B and plasma cell infiltrates in ASyS muscle and elevated levels of B cell homing factors in blood and muscle. In addition, the shift in the B cell compartment is contextualized by increased levels of soluble B and T cell factors as well as complement factors. Corroborating previous reports of elevated BAFF levels in anti-Jo-1-antibody mediated IIM [37], B cell activation and proliferation factors (APRIL, BAFF, IL-4, IL-6, IL-13, IL-21, sCD40L) capable of inducing high B cell turnover were elevated in ASyS [6, 17, 44]. Interestingly, BAFF levels were previously shown to correlate with antibody levels and readouts of disease severity. Further, we also observed subtle alterations of the T helper/regulatory compartment supplementing the peripheral immune response in ASyS. Thus, findings in peripheral blood are reminiscent of those in muscle pathology and might support other diagnostic parameters.

In further support of a B cell-mediated pathology, rituximab, a B cell-depleting anti-CD20 antibody, demonstrated clinical improvement predominantly in in Jo-1^+^ and Mi-2 myositis [1]. Further, anti Jo-1 levels in serum decreased after B cell depletion and were associated with amelioration of disease activity [2]. However, rituximab reduced anti-Jo-1 titers only by approximately 30%, which is most probably related to the persistence of memory plasma cells, which are not affected by rituximab. Thus, therapies directed against plasma cells or factors defining the B cell/plasma cell niche such as BAFF/APRIL antagonists might be more effective in ASyS [25, 33].

A limitation to this work is potentially introduced by clinical and therapeutic heterogeneity among patients and subgroups. As such, immunomodulatory treatments might introduce a potential confounder in our analysis. To address this bias, we compared patients treated with glucocorticoids to those without. Here, we observed no meaningful differences between the two groups. ASyS is a disorder that is both rare and heterogenous and standardized treatment approaches are currently lacking. To address this concern, we aimed to recruit an informative and sizable cohort from multiple centers. In addition, we are aware that other types of ASyS do exist where muscle symptoms are not prevalent; however, this study aimed at precisely describing skeletal muscle immune features. The scope of this study was, therefore, limited to the study of skeletal muscles and peripheral blood. Whether we may find a relevant B cell pathology in other organs involved in this disease such as the skin or the lungs, and whether the local micro milieu is similar to that in affected muscles remains to be elucidated.

In conclusion, our study provides evidence for a characteristic micro milieu that is particularly suited for B and plasma cells in ASyS-associated myositis, thereby offering the conceptual framework for potential B cell and plasma cell-targeting therapies in ASyS.

## Supplementary Information

Below is the link to the electronic supplementary material.

Supplementary file1 (PDF 1335 KB)
