# Supplementary material for: Skeletal muscle provides the immunological micro-milieu for specific plasma cells in anti-synthetase syndrome-associated myositis
Source: Acta Neuropathol. 2022 May 25;144(2):353–72. doi: 10.1007/s00401-022-02438-z (PMC9288384; doi:10.1007/s00401-022-02438-z)
Supplement: Supplementary file 1 — Supplementary file1 (PDF 1335 KB) [file 401_2022_2438_MOESM1_ESM.pdf]

Supplemental Table 1: Staining panels for flow cytometry

	FITC	PE	ECD	PC5.5	PC7	APC	A700	A750	BV421	BV510
**PANEL 1**	CD1c (AD5-8E7)	CD69 (TP1.55.3)	HLA-DR (Immu-357)	CD3 (UCHT1)	CD56 (N901)	CD4 (13B8.2)	CD19 (J3-119)	CD16 (3G8)	CD8 (B9.11)	CD14 (M5E2)
**PANEL 2**	IgD (IA6-2)	CD80 (L307.4)	CD24 (ALB9)	CD38 (HIT2)	CD27 (1A4CD27)	CD23 (EBVCS-5)	CD19 (J3-119)	CD20 (2H7)	CD21 (HB5)	IgM (MHM-88)
**PANEL 3**	CD183 (G025H7)	CD52 (REA164)	CD8 (SFCI21Thy 2D3)	CD194 (L29IH4)	CD196 (11A9)	CD56 (AF12-7H3)	CD14 (M5E2)	CD3 (HIT3a)	CD45RO (UCHL1)	CD4 (13B8.2)
**PANEL 4**	Helios (22F6)	FoxP3 (PCH101)	CD4 (SFCI12T4D 11)	CTLA-4 (14D3)	CD25 (B1.49.9)	CD56 (AF12-7H3)	CD8 (B9.11)	CD3 (HIT3a)	CD45RO (UCHL1)	CD127 (A019D5)
**PANEL 5**	CD45RA (ALB11)	CD57 (QA17A04)	CD31 (WM59)	CD56 (N901)	CD27 (1A4CD27)	CD226 (11A8)	CD45RO (UCHL1)	CD3 (HIT3a)	CD8 (B9.11	CD4 (13B8.2)
**PANEL 6**	IFN-γ (4S.B3)	IL-17A (eBio64CA P17)	CD4 (SFCI12T4D 11)	CD45RO (UCHL1)		CD56 (AF12-7H3)	CD8 (B9.11)	CD3 (HIT3a)	GM-CSF (BVD2-21C11)	CD146 (P1H12)
**PANEL 7**	TNF-α (Mab11)	IL-22 (22URTI)	CD4 (SFCI12T4D 11)	CD45RO (UCHL1)		CD56 (AF12-7H3)	CD8 (B9.11)	CD3 (HIT3a)	IL-6 (MQ2-13A5)	CD146 (P1H12)

Supplemental Table 2: Gating hierarchy used for flow cytometric analyses

- Lymphocytes: CD45+SSClowCD14-
  - T cells: CD3+CD56-
    - CD4+ T cells: CD4+CD8-
      - Naïve: CD45RA+CD27+
      - CM: CD45RA-CD27+
      - EM: CD45RA-CD27-
      - RTE: CD45RA+CD31+
      - TH1: CD45RO+CCR4-CCR6-CXCR3+
      - TH2: CD45RO+CCR4+CCR6-CXCR3-
      - TH17: CD45RO+CCR4+CCR6+CXCR3-
      - Treg: CD45RO+CD127lowFoxP3+CD25+
      - TNF-α
      - IFN-γ
      - IL-17
    - CD8+ T cells: CD8+CD4-
      - Naïve: CD45RA+CD27+
      - CM: CD45RA-CD27+
      - EM: CD45RA-CD27-
      - TEMRA: CD45RA+CD27-
      - TNF-α
      - IFN-γ
      - IL-17
  - B cells: CD19highCD3-
    - Naïve: CD20+CD27-IgD+IgM+
    - Class-switch: CD20+CD27+IgD-IgM-
    - IgM only: CD20+CD27+IgD-IgM+
    - Marginal-zone like: CD20+CD27+IgD+IgM+
    - Transitional: CD20+IgD+IgM+CD38+CD21+CD24+
  - Plasma cells: CD19lowCD138high
  - NK cells: CD56+CD3-
  - NKT cells: CD3+CD56+
  - Monocytes: CD45+SSCmidCD14+
  - Granulocytes: CD45+SSChighCD14-

Supplemental Table 3: Multiplex analysis of complement factors.

Protein Accessions	Genes	Protein Descriptions
**P07358**	C8B	Complement component C8 beta chain
**P02747**	C1QC	Complement C1q subcomponent subunit C
**Q07021**	C1QBP	Complement component 1 Q subcomponent-binding protein, mitochondrial
**P05156**	CFI	Complement factor I
**P00751**	CFB	Complement factor B
**P02748**	C9	Complement component C9
**P08603**	CFH	Complement factor H
**P01024**	C3	Complement C3
**P0C0L4**	C4A	Complement C4-A
**P07360**	C8G	Complement component C8 gamma chain
**P01031**	C5	Complement C5
**P02746**	C1QB	Complement C1q subcomponent subunit B

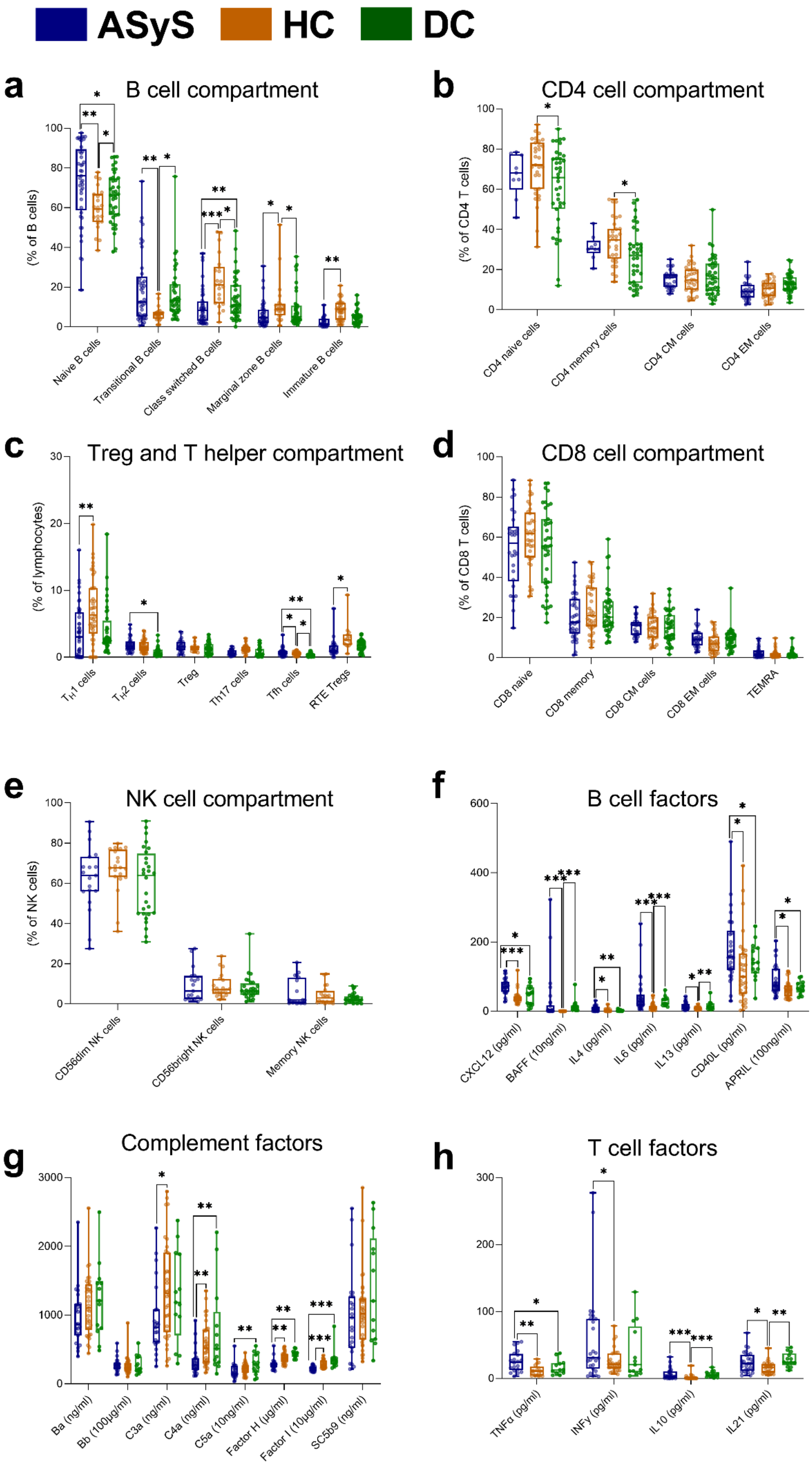

ASyS
HC
DC
a
B cell compartment
(% of B cells)
Naive B cells
Transitional B cells
Class switched B cells
Marginal zone B cells
Immature B cells
b
CD4 cell compartment
(% of CD4 T cells)
CD4 naive cells
CD4 memory cells
CD4 CM cells
CD4 EM cells
c
Treg and T helper compartment
(% of lymphocytes)
T_H1 cells
T_H2 cells
Treg
Th17 cells
Tfh cells
RTE Tregs
d
CD8 cell compartment
(% of CD8 T cells)
CD8 naive
CD8 memory
CD8 CM cells
CD8 EM cells
TEMRA
e
NK cell compartment
(% of NK cells)
CD56dim NK cells
CD56bright NK cells
Memory NK cells
f
B cell factors
CXCL12 (pg/ml)
BAFF (10ng/ml)
IL4 (pg/ml)
IL6 (pg/ml)
IL13 (pg/ml)
CD40L (pg/ml)
APRIL (100ng/ml)
g
Complement factors
Ba (ng/ml)
Bb (100µg/ml)
C3a (ng/ml)
C4a (ng/ml)
C5a (10ng/ml)
Factor H (µg/ml)
Factor I (10µg/ml)
SC5b9 (ng/ml)
h
T cell factors
TNFα (pg/ml)
INFγ (pg/ml)
IL10 (pg/ml)
IL21 (pg/ml)

Supp. Figure 1: Individual immune compartments in ASyS. In-depth analysis of cellular changes in ASyS patients (n = 36) compared to HC (n = 40) and DC (n = 40) were performed by flow cytometric analysis. The B cell (**a**), CD4 cell (**b**), Treg and T helper (**c**), CD8 cell (**d**) and NK cell compartment were analyzed. Soluble factors were analyzed by multiplex immunoassay and include B cell (**f**), complement (**g**) and T cell factors (**h**). The Kruskal-Wallis one-way analysis of variance with Dunn's multiple comparison test was used. *p<0.05, **p<0.01, ***p<0.001. *Abbreviations: ASyS = Anti-synthetase syndrome; BAFF = B-cell activating factor; CM = central memory; DC = diseased control; EM = effector memory; HC = healthy control; IL = interleukin; TEMRA = effector memory T cells re-expresses CD45RA; Tfh = T follicular helper; Th = T helper; RTE = recent thymic emigrates.*

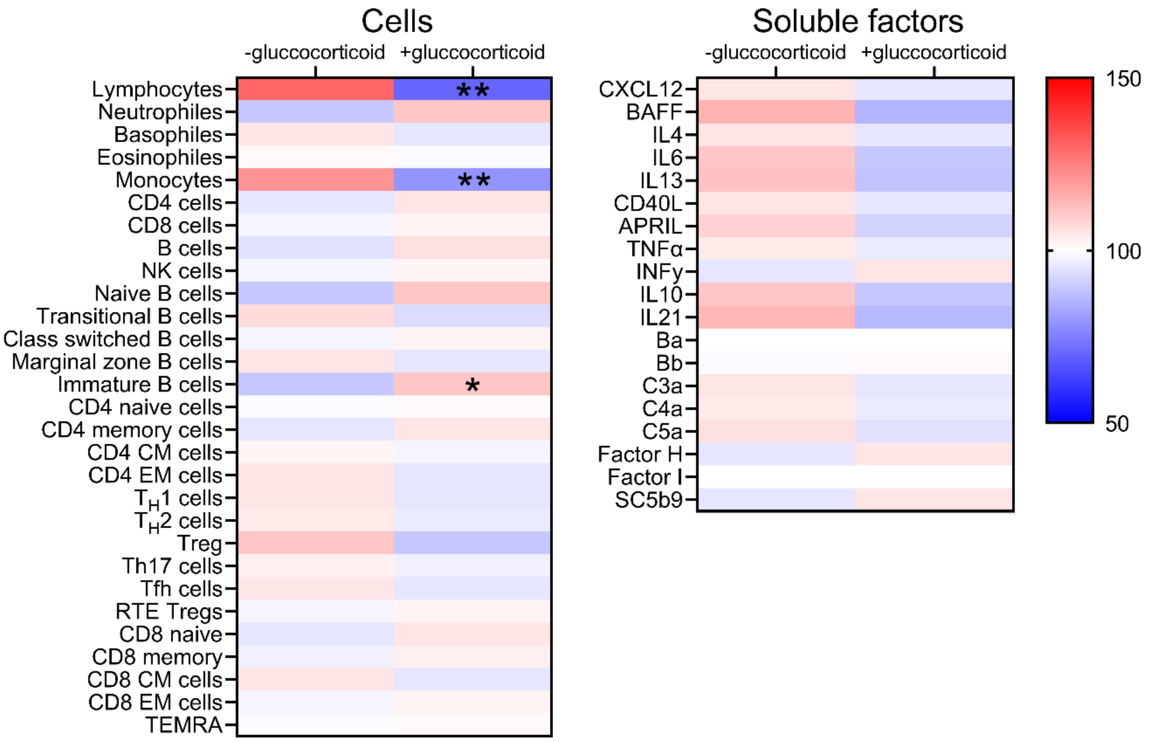

Supp. Fig. 2: Comparison of glucocorticoid treatment in ASyS. We compared ASyS patients with glucocorticoid treatment (n = 17) to those without (n =19). Features of the peripheral immune response were analyzed. Statistical analysis was performed using the Mann-Whitney U test (unpaired comparisons). The level of significance was set to $p < 0.05$. *p<0.05, **p<0.01. *Abbreviations: ASyS = Anti-synthetase syndrome; BAFF = B-cell activating factor; CM = central memory; DC = diseased control; EM = effector memory; HC = healthy control; IL = interleukin; TEMRA = effector memory T cells re-expresses CD45RA; Tfh = T follicular helper; Th = T helper; RTE = recent thymic emigrates.*

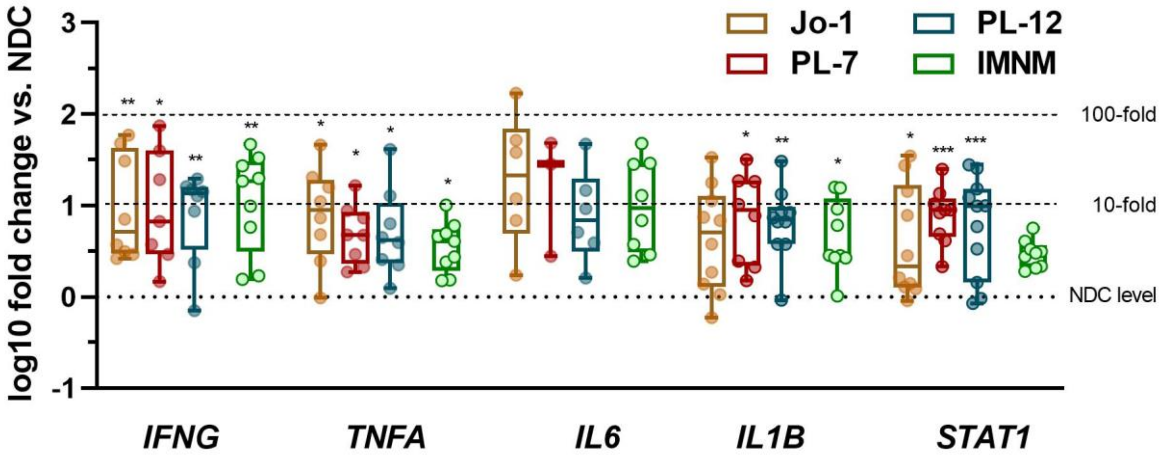

Supp. Figure 3: Expression levels of type II interferon genes in ASyS. Box plots displaying the log10 fold change of indicated gene expression (*IFNG*, *TNFA*, *IL6*, *IL1B*, *STAT1*) in ASyS (Jo-1 n=6, PL7 n=9, PL-12 n=8) subgroups and DC (IMNM n=9) compared to NDCs. Significant differences between ASyS, DC and NDCs are indicated by significance levels above each comparison. Kruskal-Wallis one-way analysis of variance with Dunn multiple comparison test. The level of significance was set to $p < 0.05$. *p<0.05, **p<0.01, ***p<0.001.

Of note, most PL-7+ patients had undetectable level of *IL6* and fold-change could not be calculated, hence only three dots are displayed.

Abbreviations: ASyS = Anti-synthetase syndrome; DC = disease control; IFNG = interferon γ; IL = interleukin; IMNM = immune-mediated necrotizing myopathy; NDC = non-diseased control.

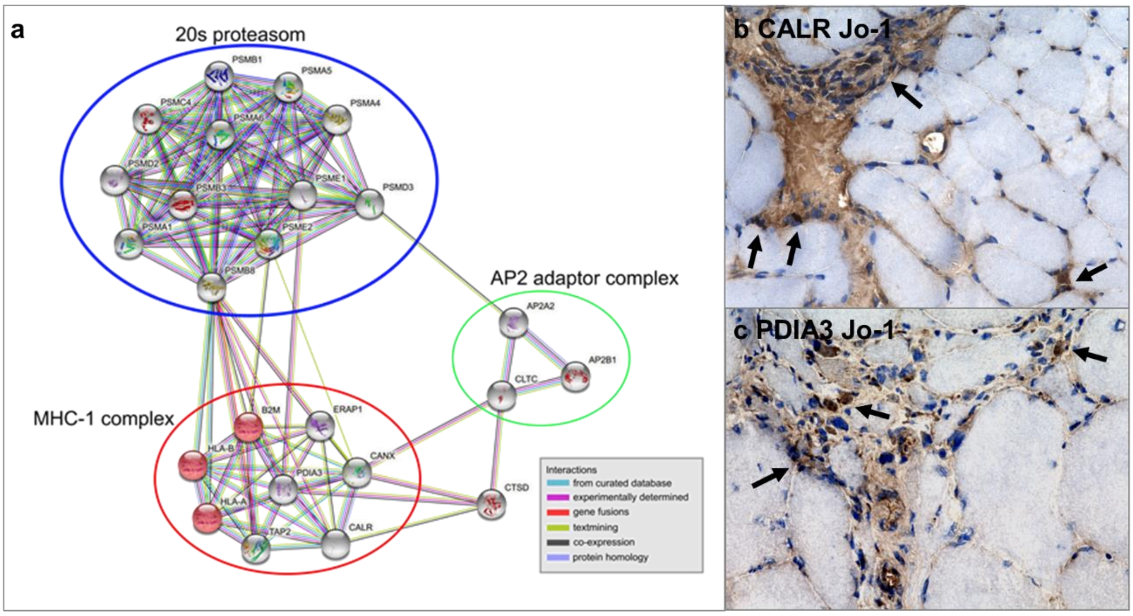

Supp. Figure 4: (a) Network analysis of antigen processing antigen presentation and processing using STRING identified the 20s proteasom, the MHC-1 complex and the AP2 adaptor complex in the ASyS proteom. Immunohistochemical analysis of CALR (**b**) and PDIA3 (**c**) belonging to the MHC-1 complex. *Abbreviations: ASyS = Anti-synthetase syndrome; CALR = calreticulin; PDIA3 = Protein Disulfide Isomerase Family A Member 3; STRING = Search Tool for the Retrieval of Interacting Genes/Proteins.*

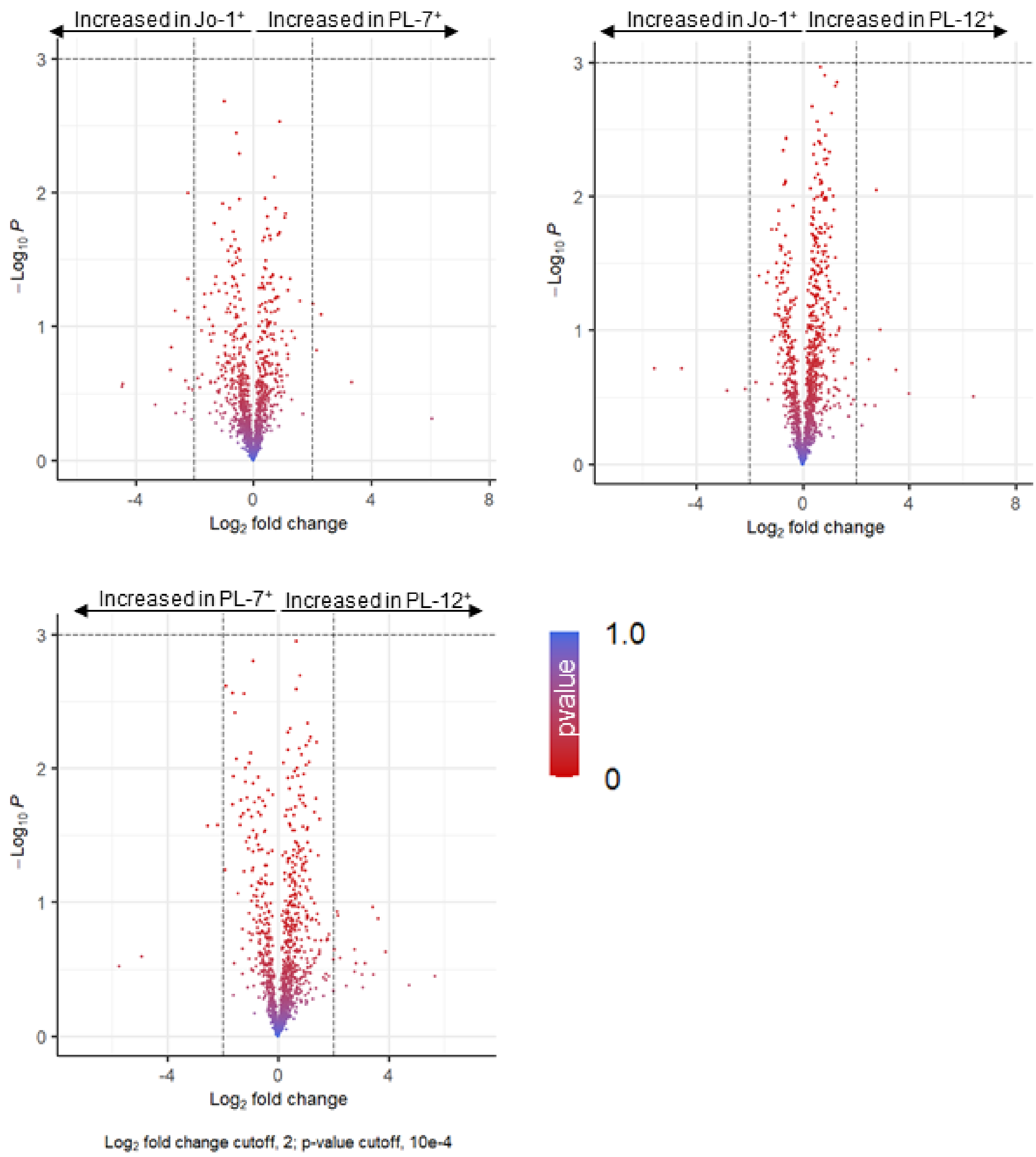

Supp. Figure 5: Proteome profiles of serological ASyS subgroups. Proteomic profiling of muscle biopsies from ASyS patients comparing different ASyS subgroups (anti-Jo-1⁺ n = 9, anti-PL-7⁺ n = 7, anti-PL-12⁺ n = 4) displayed as volcano plots. Volcano plots were constructed by calculating the $\log_2$ fold change of the median and the $-\log_{10}$ p-value. The $\log_2$ fold change and p-value cutoff are indicated.

Gene set enrichment analysis for biological processes, cellular components and molecular functions displayed no meaningful differences (data not shown). The significance level was set to p (FDR) < 0.05. Statistical analysis was performed using the Mann-Whitney U test (unpaired comparisons).

Abbreviations: ASyS = Anti-synthetase syndrome; FDR = false discovery rate.

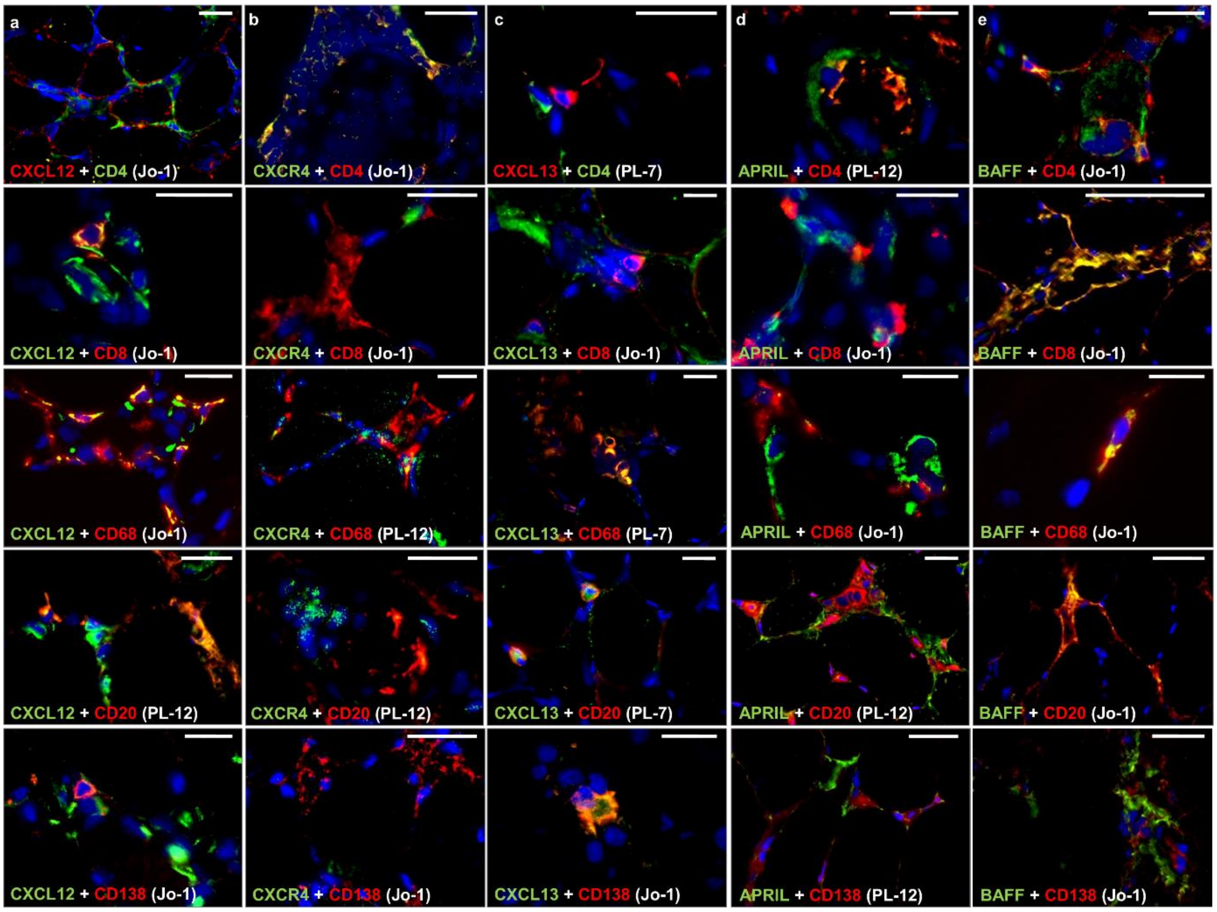

Supp. Figure 6: Protein expression of BAFF within B cells, but not plasma cells, APRIL is not expressed by either cell type. Double immuno fluorescence staining reveals co-labelling of homing factors CXCL12 (**a**) and CXCL13 (**c**) with multiple cell types, CXCR4 co-labels with T cells (**b**), while APRIL is not expressed from any of these cells (**d**). BAFF is expressed by T cells, as well as macrophages and B cells, but not plasma cells (**e**).

ASyS = Anti-synthetase syndrome; BAFF = B-cell activating factor.

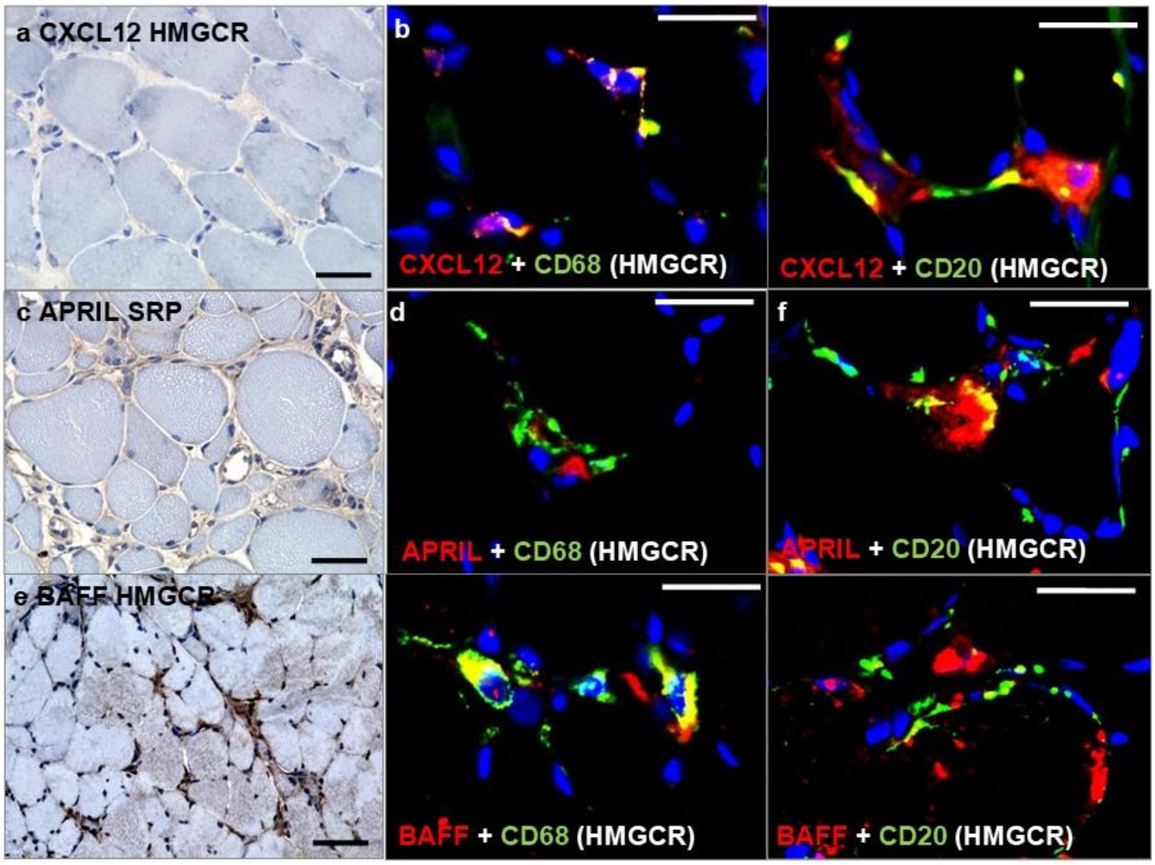

Supp. Figure 7: Protein expression of interesting factors in DC patients. Histological staining of CXCL12, APRIL and BAFF in IMNM patients is shown (a, c, e), while double immuno fluorescence staining reveals co-labelling of these factors with multiple cell types.

IMNM = immune-mediated necrotizing myopathy
